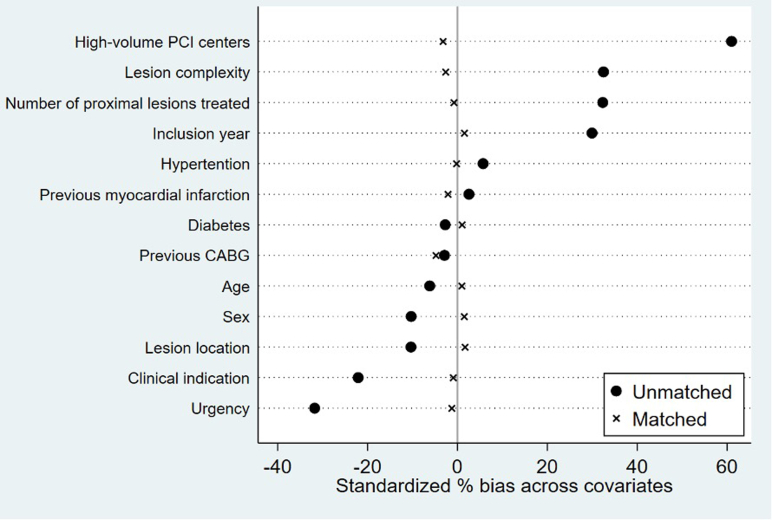# Intracoronary Imaging of Proximal Coronary Artery Lesions **–** A Nationwide Lesion-Level Analysis From SCAAR

**DOI:** 10.1016/j.jscai.2023.100597

**Published:** 2023-02-20

**Authors:** Sacharias von Koch, Sofia Bergman, Pontus Andell, Göran K. Olivecrona, Matthias Götberg, Elmir Omerovic, Ole Fröbert, Sergio Buccheri, Stefan James, Sasha Koul, Moman A. Mohammad, David Erlinge

**Affiliations:** a Department of Cardiology, Clinical Sciences, Lund University, Skåne University Hospital, Lund, Sweden; b Heart and Vascular Theme, Karolinska University Hospital, and Unit of Cardiology, Department of Medicine, Karolinska Institutet, Stockholm, Sweden; c Department of Cardiology, Sahlgrenska University Hospital, Gothenburg, Sweden; d Örebro University, Faculty of Health, Department of Cardiology, Örebro, Sweden and Department of Clinical Medicine, Aarhus University Health, Aarhus, Denmark; e Department of Medical Sciences, Uppsala University, and Uppsala Clinical Research Center, Uppsala, Sweden; f Division of Cardiology, University of California San Diego, San Diego, California

**Keywords:** intracoronary imaging, intravascular ultrasound, optical coherence tomography, target lesion revascularization

## Abstract

**Background:**

Current evidence suggests that use of intracoronary imaging during percutaneous coronary intervention (PCI) of the left main coronary artery (LMCA) reduces mortality. However, there is a scarcity of data on the overall role of intracoronary imaging, particularly in other non-LMCA proximal coronary artery lesions. We aimed to investigate the association of use of intracoronary imaging on outcome in proximal lesions treated with PCI.

**Methods:**

The Swedish Coronary Angiography and Angioplasty Registry was used to identify all proximal coronary artery lesions treated with stent implantation between June 11, 2013, and January 16, 2021. Proximal coronary artery lesions (LMCA, proximal left anterior descending artery, left circumflex artery, and right coronary artery) assessed by intracoronary imaging before and/or after stent implantation were matched to control lesions treated based on angiography alone using propensity score matching. The primary end point was target lesion revascularization with PCI, and secondary end points included all-cause mortality and definite stent thrombosis within 3 years.

**Results:**

Among the 3623 matched pairs, intracoronary imaging was associated with significantly lower risk of target lesion revascularization, 3.7% vs 4.7%; hazard ratio (HR), 0.77; 95% CI, 0.61-0.97; *P* = .025, and all-cause mortality, 9.1% vs 12.8%; HR, 0.70; 95% CI, 0.61-0.81; *P* < .001, with no difference in definite stent thrombosis.

**Conclusions:**

The use of intracoronary imaging in proximal coronary artery lesions is associated with lower rates of repeat revascularization and better survival. The results appear to be primarily driven by improved outcome of LMCA lesions. These results reinforce the role of intracoronary imaging in assessing and treating proximal coronary lesions.

## Highlights

• This study on intracoronary imaging of proximal lesions comprises 3623 propensity matched pairs.
• Intracoronary imaging was associated with lower rates of target lesion revascularization and better survival.
• Results appear to be primarily driven by improved outcome of left main coronary artery lesions.
• Centers with a frequent use of intracoronary imaging had lower rates of TLR.

## Central Illustration

**Figure:**
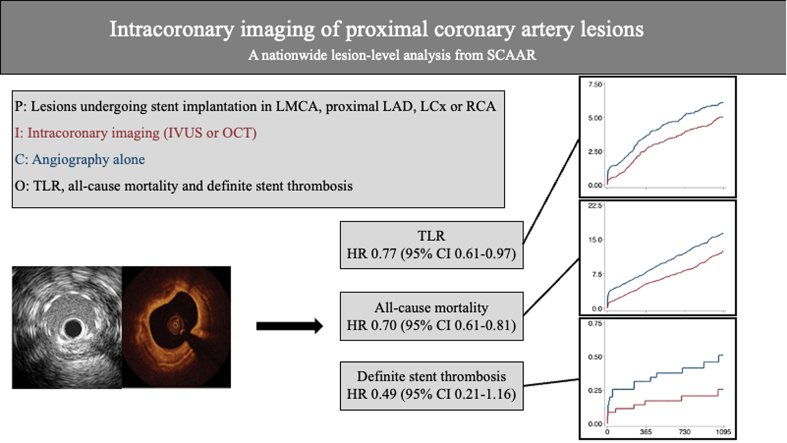

## Introduction

Standard x-ray coronary angiography provides a 2-dimensional assessment of coronary vessel anatomy and diameter that is far less than optimal for characterizing the often complex 3-dimensional structure of the coronary arteries. Intravascular ultrasound (IVUS) or optical coherence tomography (OCT) are 2 intracoronary imaging modalities that can overcome the limitations of coronary angiography. Intracoronary imaging provides additional information that can help optimize stent sizing, expansion, and apposition and, in turn, reduces the risk of stent failure.^1^^,^^2^ In large randomized clinical trials, the use of intracoronary imaging to optimize stent implantation has been demonstrated to reduce the risk of major adverse cardiovascular events and target lesion revascularization (TLR).^3^^,^^4^ Meta-analyses, including smaller neutral clinical trials as well as observational studies, show a similar association.^5^, ^6^, ^7^, ^8^, ^9^ In the current European guidelines, intracoronary imaging receives a class 2, level A recommendation for use in selected patients to optimize stent implantation.^10^ However, except for left main coronary artery (LMCA) lesions, there are little data supporting the selection of patients and lesions for intracoronary imaging.

Using the nationwide Swedish Coronary Angiography and Angioplasty Registry (SCAAR), we conducted a lesion-level analysis investigating the association between intracoronary imaging guided stent implantation and outcomes for proximal lesions in the left anterior descending (LAD) artery, left circumflex (LCx) artery, and right coronary artery (RCA).

## Methods

### Data source

We conducted a nationwide registry-based retrospective cohort study using SCAAR, a subregistry of the Swedish Web-system for Enhancement and Development of Evidence-based care in Heart disease Evaluated According to Recommended Therapies (SWEDEHEART).^11^ SCAAR includes all patients undergoing coronary angiography and/or percutaneous coronary intervention (PCI) in Sweden. The registry contains extensive information including patient characteristics (eg, sex, age, comorbidities) and procedure information (eg, stent characteristics, segments stented, lesion characteristics). In June 2013, the registry introduced a new intracoronary diagnostic module to collect more detailed information about the use of intracoronary imaging and anatomical and physiological diagnostic procedures.

### Study design, study population, and outcome

**Figure 1:**
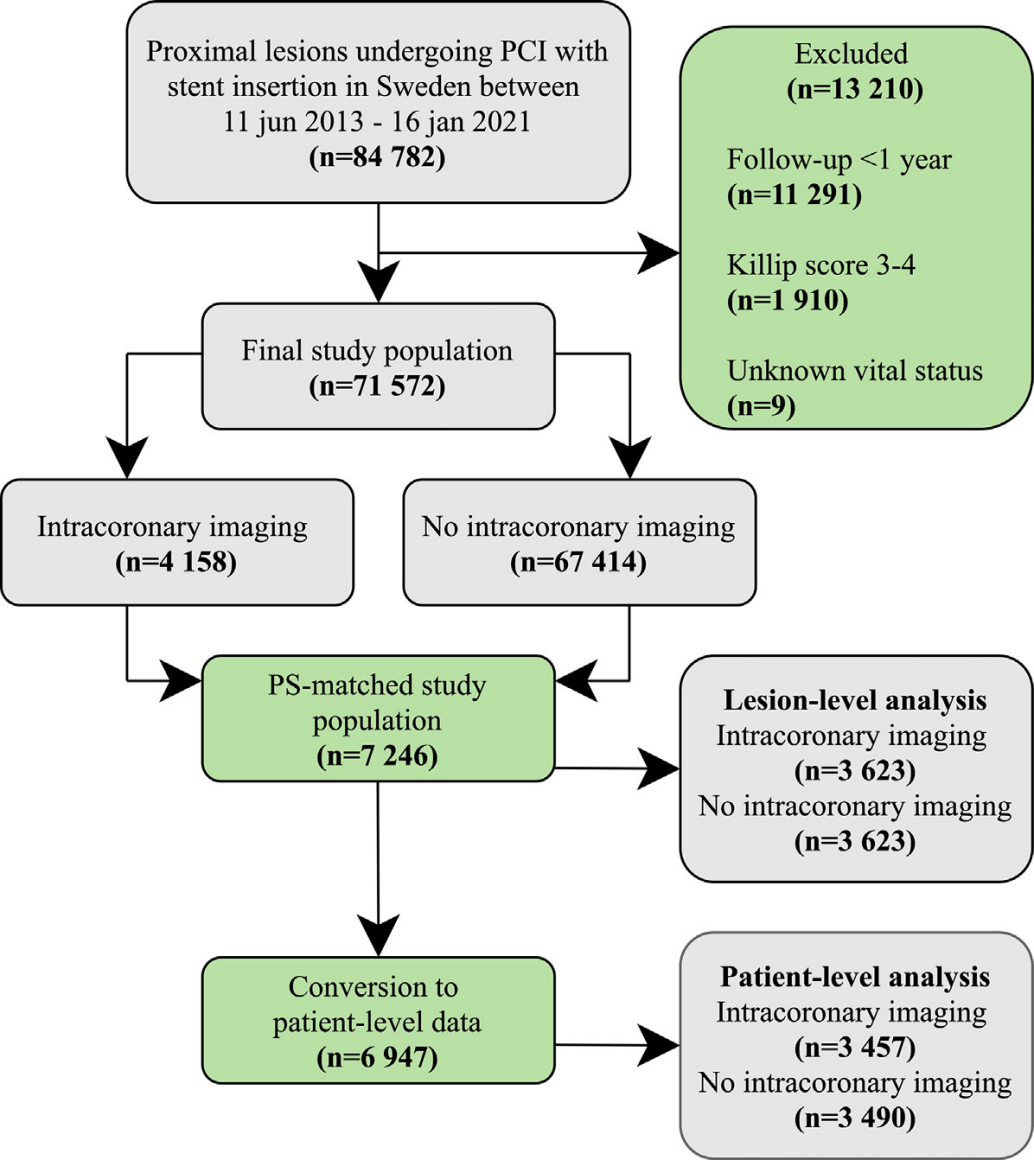
**Study flow chart**, Lesions undergoing successful PCI with stent implantation in a proximal coronary artery segment were included and stratified by use of intracoronary imaging or no intracoronary imaging during procedure. One-to-one propensity score matching based on sex, age, inclusion year, clinical indication for PCI, high-volume intracoronary imaging PCI centers, number of proximal lesions treated, lesion location, diabetes, hypertension, urgency, lesion complexity, previous myocardial infarction, and previous coronary artery bypass surgery. A total of 7246 lesions were included. PCI, percutaneous coronary intervention.

The study adhered to the Strengthening the Reporting of Observational Studies in Epidemiology (STROBE) guidelines and was approved by the Regional Ethical Review Board in Lund, Sweden (approval number 2015/297). The objective of this study was to assess the association of intracoronary imaging usage in proximal coronary artery lesions and outcomes after stent implantation. We hypothesized that intracoronary imaging optimizes stent implantation in proximal arteries and therefore results in lower rates of stent failure, translating into lower mortality. We identified all stented proximal lesions in SCAAR between June 11, 2013, and January 16, 2021, and using the modified American Heart Association classification of segmental anatomy, we included all proximally treated lesions: LMCA (segment 5), LAD (segment 6), LCx (segment 11), and RCA (segment 1). The exclusion criteria and flowchart are presented in Figure 1. Coronary artery lesions were stratified according to use of intracoronary imaging with IVUS, OCT, or none before or after stent implantation to guide PCI. The primary end point was TLR within 3 years of the index procedure. Secondary end points included all-cause mortality and definite stent thrombosis, defined according to Academic Research Consortium criteria.^12^ Propensity score (PS) matching was performed to adjust for confounding. One assumption of survival analysis includes independence of observations. The lesion-level analysis assumes independence of lesions as separate observations having unique outcome data, which are target lesion revascularization and stent thrombosis. In the lesion-level analysis, lesions originating from the same patient could therefore be represented in both groups. However, all-cause mortality was assessed using patient-level data to adhere to the assumption of independence. Patients were included in the intracoronary imaging group if IVUS or OCT was used during PCI in at least one proximal lesion. Prespecified subgroup analyses stratified for sex (male vs female), age (younger than 75 vs older than 75 years), diabetes mellitus (diabetes vs no diabetes), indication (chronic coronary syndrome vs acute coronary syndrome), high-volume PCI center (PCI centers with >2500 stented proximal lesions vs PCI centers with ≤2500 stented proximal lesions), high-volume intracoronary imaging centers (PCI centers with more than 5% intracoronary imaging vs PCI centers with less than 5% intracoronary imaging), urgency (elective vs subacute and acute interventions), stent diameter (<4.00 mm vs ≥4.0 mm), lesion location (LMCA vs other proximal lesions), coronary artery bypass graft (coronary artery bypass graft vs no coronary artery bypass graft) and lesion complexity (Type A or B1-B2 vs Type C or B1-B2 with bifurcation) were done for intracoronary imaging including both IVUS/OCT as well as IVUS and OCT assessed separately. A sensitivity analysis investigating IVUS and OCT was also done. Finally, a segment stratification analysis was carried out.

### Statistical analyses

Continuous data are presented as medians with 25th and 75th percentiles, and categorical data are presented as counts and percentages. Differences in continuous variables between the groups were calculated with independent *t* test, and differences between categorical data were calculated with the χ^2^ test. One-to-one PS matching was performed to match the intracoronary imaging group with controls from the nonintracoronary imaging group. Lesions were matched using the nearest-neighbor method with caliper width 0.1 based on the following variables: sex, age, inclusion year, clinical indication for coronary angiography and PCI (chronic coronary syndrome, unstable angina pectoris, non-ST-elevation myocardial infarction, ST-elevation myocardial infarction, and other), high-volume intracoronary imaging PCI centers, the total number of proximal lesions treated, lesion location (LMCA, proximal LAD, proximal LCx, and proximal RCA), diabetes, hypertension, previous myocardial infarction, urgency (elective, subacute, and acute intervention), previous CABG surgery, and lesion complexity. These variables were identified a priori based on previous literature and clinical experience. Differences in outcome were analyzed by estimating the event rates with Kaplan-Meier estimator and univariable Cox proportional hazard models presented as hazard ratio (HR) along with 95% CI. Lesion-level data was used for assessment of TLR and ST, treating each lesion as a unique observation having a unique outcome. However, to minimize eventual bias related to this assumption, we used a multilevel hierarchical Cox regression model with lesions nested on the patient level. The proportion of missing values for all variables of interest were <1% (Supplementary Table 2 and Table 1), and all analyses were therefore done on complete case data. A 2-sided *P* value less than .05 was considered statistically significant. All statistical analyses were performed in STATA SE (version 16.1; StataCorp).

**Table 1:** Baseline characteristics for the propensity score-matched study population.

	Intracoronary imaging (n = 3623)	No intracoronary imaging (n = 3623)	*P*	Missing
**Demographic characteristics**				
Age, y	68.6 [68.3-69.0]	68.7 [68.3-69.0]	.92	0.0
Age >75	1028 (28.4)	982 (27.1)	.23	0.0
Male	2809 (77.5)	2832 (78.2)	.52	0.0
Current smoker	512 (14.9)	534 (15.5)	.72	4.9
**Comorbidities**				
Diabetes mellitus	782 (21.6)	767 (21.2)	.67	0.0
Hypertension	2459 (67.9)	2463 (68.0)	.92	0.0
Previous myocardial infarction	898 (24.8)	931 (25.7)	.37	0.0
Previous PCI	961 (26.5)	888 (24.5)	.05	0.0
Previous CABG	240 (6.6)	283 (7.8)	.05	0.0
Hyperlipidemia	2150 (59.5)	2082 (57.7)	.12	0.3
Estimated GFR, mL/min	80.7 [79.8-81.5]	81.1 [80.1-81.9]	.53	15.3
Chronic kidney disease stage			.52	15.4
Stage I–II	2549 (82.0)	2491 (82.5)		
Stage III	487 (15.7)	450 (14.9)		
Stage IV–V	71 (2.3)	79 (2.6)		
**In-hospital characteristics**				
Inclusion time			.12	0.0
Early (2013–2015)	992 (27.4)	1060 (29.3)		
Mid (2016–2018)	1840 (50.8)	1759 (48.6)		
Late (2019–2021)	791 (21.8)	804 (22.2)		
High-volume intracoronary imaging PCI center	2839 (78.4)	2892 (79.8)	.13	0.0
Indication			.94	0.0
Chronic coronary syndrome	1056 (29.2)	1036 (28.6)		
Unstable angina	625 (17.3)	615 (17.0)		
NSTEMI	1230 (34.0)	1265 (34.9)		
STEMI	438 (12.1)	435 (12.0)		
Other	274 (7.6)	272 (7.5)		
Urgency			.30	0.0
Elective	1327 (36.6)	1277 (35.3)		
Subacute	1710 (47.2)	1776 (49.0)		
Acute	586 (16.2)	570 (15.7)		
Killip class at presentation			.95	12.1
1	3118 (96.6)	3037 (96.6)		
2	110 (3.4)	108 (3.4)		
**Medical treatment prior to PCI or periprocedural added medical treatment**				
Dual antiplatelet therapy	3483 (96.4)	3467 (96.0)	.39	0.3
Aspirin	3551 (98.1)	3528 (97.5)	.065	0.1
Clopidogrel	1332 (36.8)	1280 (35.4)	.21	0.0
Prasugrel	20 (0.6)	15 (0.4)	.40	0.0
Ticagrelor	2264 (62.6)	2316 (64.0)	.20	0.1
Heparin	3389 (93.5)	3358 (92.7)	.16	0.0
Bivalirudin	249 (6.9)	249 (6.9)	1.00	0.0
Fondaparinux	810 (22.4)	832 (23.0)	.53	0.0
Glycoprotein IIb/IIIa inhibitor	155 (4.3)	122 (3.4)	.043	0.0
**Procedure characteristics**				
Concomitantly stented proximal segments			.82	0.0
1	2380 (65.7)	2368 (65.4)		
2	1003 (27.7)	1006 (27.8)		
3	222 (6.1)	235 (6.5)		
4	18 (0.5)	14 (0.4)		
Vascular approach			.28	0.1
Femoral	757 (20.9)	710 (19.6)		
Radial	2766 (76.5)	2808 (77.5)		
Other	93 (2.6)	105 (2.9)		
Fluoroscopy time, min	24.9 [24.4-25.5]	23.6 [23.0-24.3]	.003	0.0
Contrast volume, mL	207.4 [204.4-210.4]	189.9 [186.8-192.9]	<.001	0.0
No. of stents			.01	0.0
1	1310 (36.2)	1190 (32.9)		
2	1041 (28.7)	1069 (29.5)		
3 or more	1272 (35.1)	1364 (37.7)		
Aortic balloon pump	12 (0.3)	12 (0.3)	1.000	0.0
**Segment characteristics**				
Segments stented			.57	0.0
Left main coronary artery	1722 (47.5)	1747 (48.2)		
Proximal LAD	1446 (39.9)	1402 (38.7)		
Proximal LCx	235 (6.5)	231 (6.4)		
Proximal RCA	220 (6.1)	243 (6.7)		
ACC/AHA lesion classification			.85	0.3
Type A	124 (3.4)	118 (3.3)		
Type B1–B2	1383 (38.3)	1401 (38.9)		
Type C or B1–B2 with bifurcation	2108 (58.3)	2092 (57.9)		
Thrombus aspiration	71 (2.0)	54 (1.5)	.13	0.0
Direct stent vs balloon and stent			.08	0.0
Direct stent	609 (16.8)	555 (15.3)		
Balloon and stent	3014 (83.2)	3068 (84.7)		
Drug-eluting stent	3593 (99.5)	3585 (99.3)	.24	0.3
Stent length, mm	22.9 (22.6-23.2)	23.4 (23.1-23.7)	.03	0.0
Stent diameter, mm	4.0 (4.0-4.0)	3.6 (3.6-3.6)	<.001	0.0
Stent diameter categories			<.001	0.0
<3.00	117 (3.2)	338 (9.3)		
3.00 to <3.50	410 (11.3)	803 (22.2)		
3.50 to <4.00	950 (26.2)	1156 (31.9)		
4.00 to <4.50	982 (27.1)	745 (20.6)		
>4.50	1164 (32.1)	578 (16.0)		
Max pressure in balloon, atm	19.5 [19.4-19.6]	19.4 [19.3-19.5]	.21	0.5
Post dilatation	2991 (82.6)	2461 (67.9)	<.001	0.0

Values are median [IQR] or n (%).

ACC, American College of Cardiology; AHA, American Heart Association; CABG, coronary artery bypass grafting; GFR, glomerular filtration rate; LAD, left anterior descending coronary artery; LCx, left circumflex coronary artery; NSTEMI, non-ST-elevation myocardial infarction; PCI, percutaneous coronary intervention; RCA, right coronary artery; STEMI, ST-elevation myocardial infarction.

## Results

### Study population characteristics

After PS matching, there were 3623 lesion pairs who underwent intracoronary imaging and standard coronary angiography. After PS matching, there were no significant difference in PS between the intracoronary imaging group and the coronary angiography group (*P* = .412) (Supplementary Table 3 and Supplementary Figure 1), and no significant differences were observed in any variable used to match the 2 groups (Table 1). The median age was 68.6 years, and 77.9% of the lesions were observed in male patients. The use of intracoronary imaging was associated with significantly larger stent diameter (4.0 mm vs 3.6 mm; *P* < .001), more frequent use of post dilation (82.6% vs 67.9%; *P* < .001), and lower number of stents deployed (*P* = .01) (Table 1).

### Outcomes

**Figure 2:**
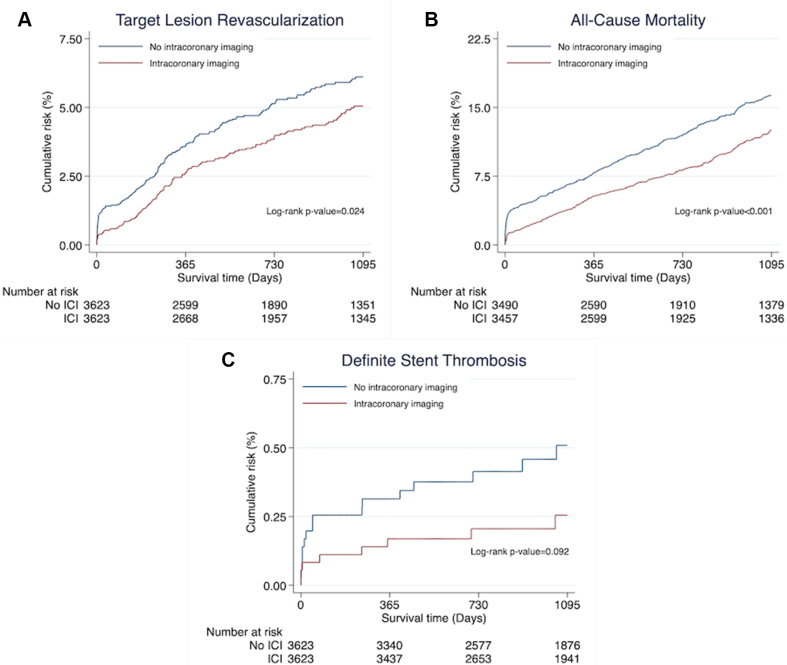
**Outcome**. Time-to-event Kaplan-Meier curves showing the event rate of target lesion revascularization (**A**), all-cause mortality (**B**), and definite stent thrombosis (**C**). ICI, intracoronary imaging.

**Figure 3:**
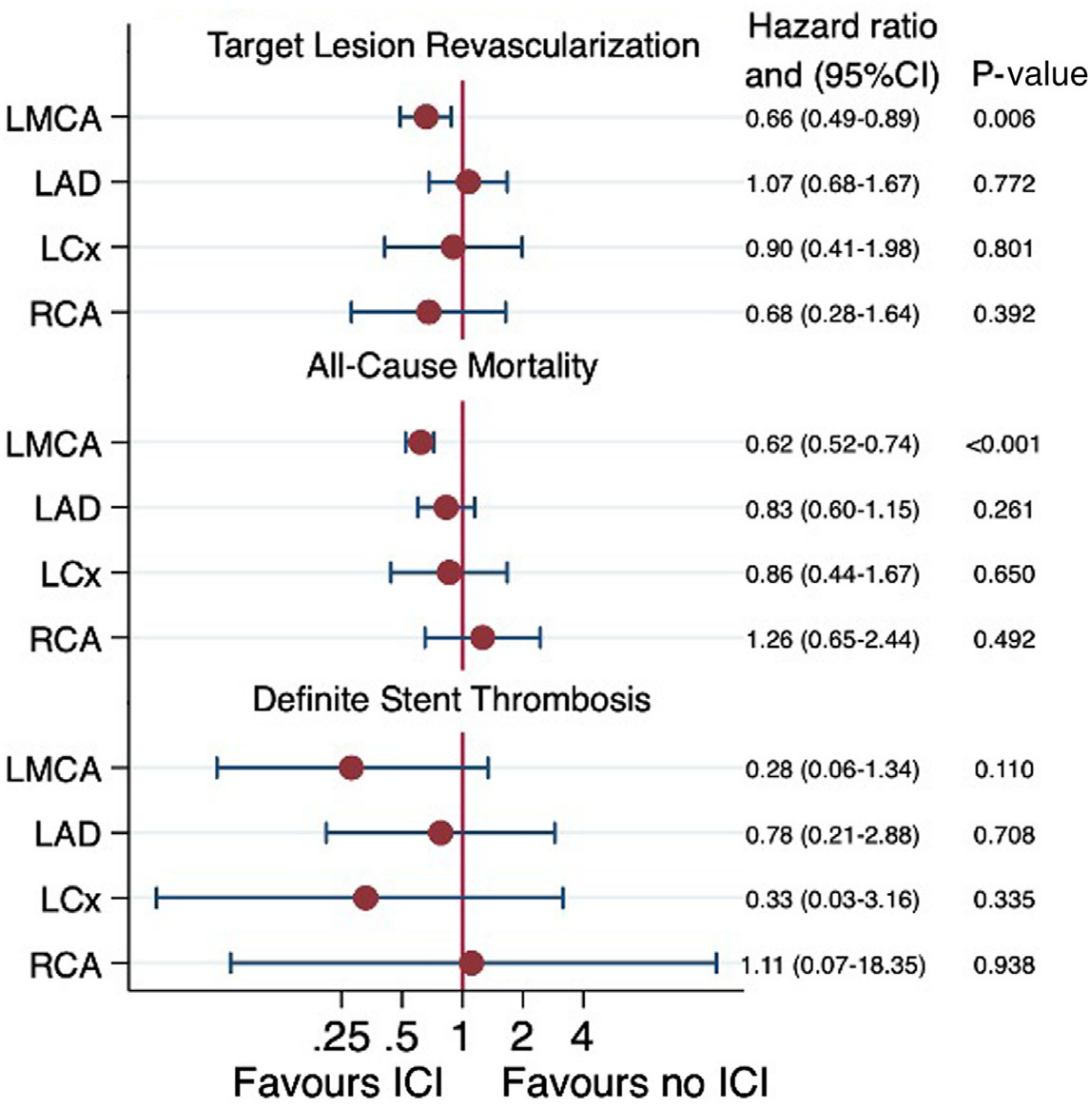
**Segment stratification.** Forest plot for segment stratification. LAD, proximal left anterior descending artery; LCx, proximal left circumflex coronary artery; LMCA, left main coronary artery; RCA, proximal right coronary artery.

**Figure 4:**
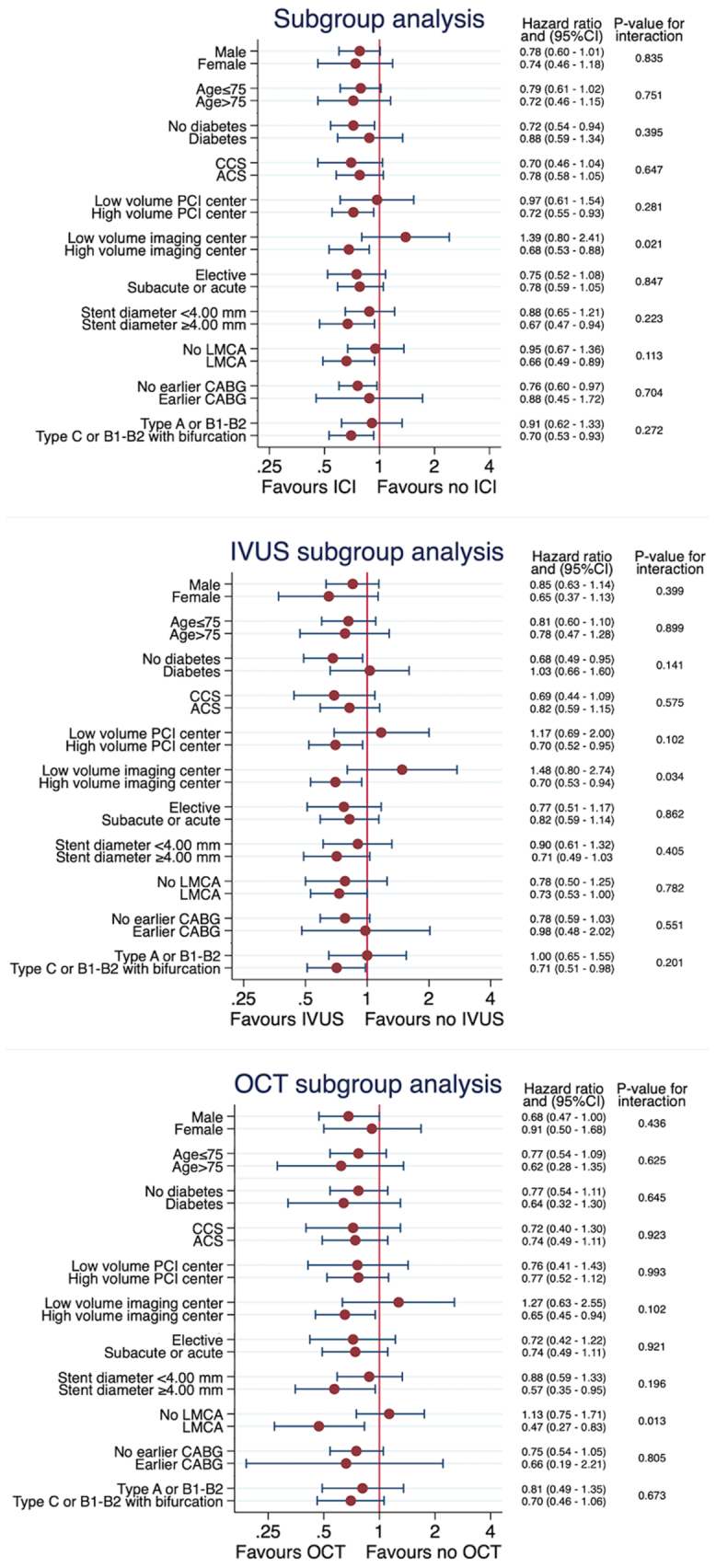
**Subgroup analysis.** Forest plot for subgroup analysis. ACS, acute coronary syndrome; CABG, coronary artery bypass graft; CAD, coronary artery disease; CCS, chronic coronary syndrome; ICI, intracoronary imaging; LMCA, left main coronary artery.

**Figure 5:**
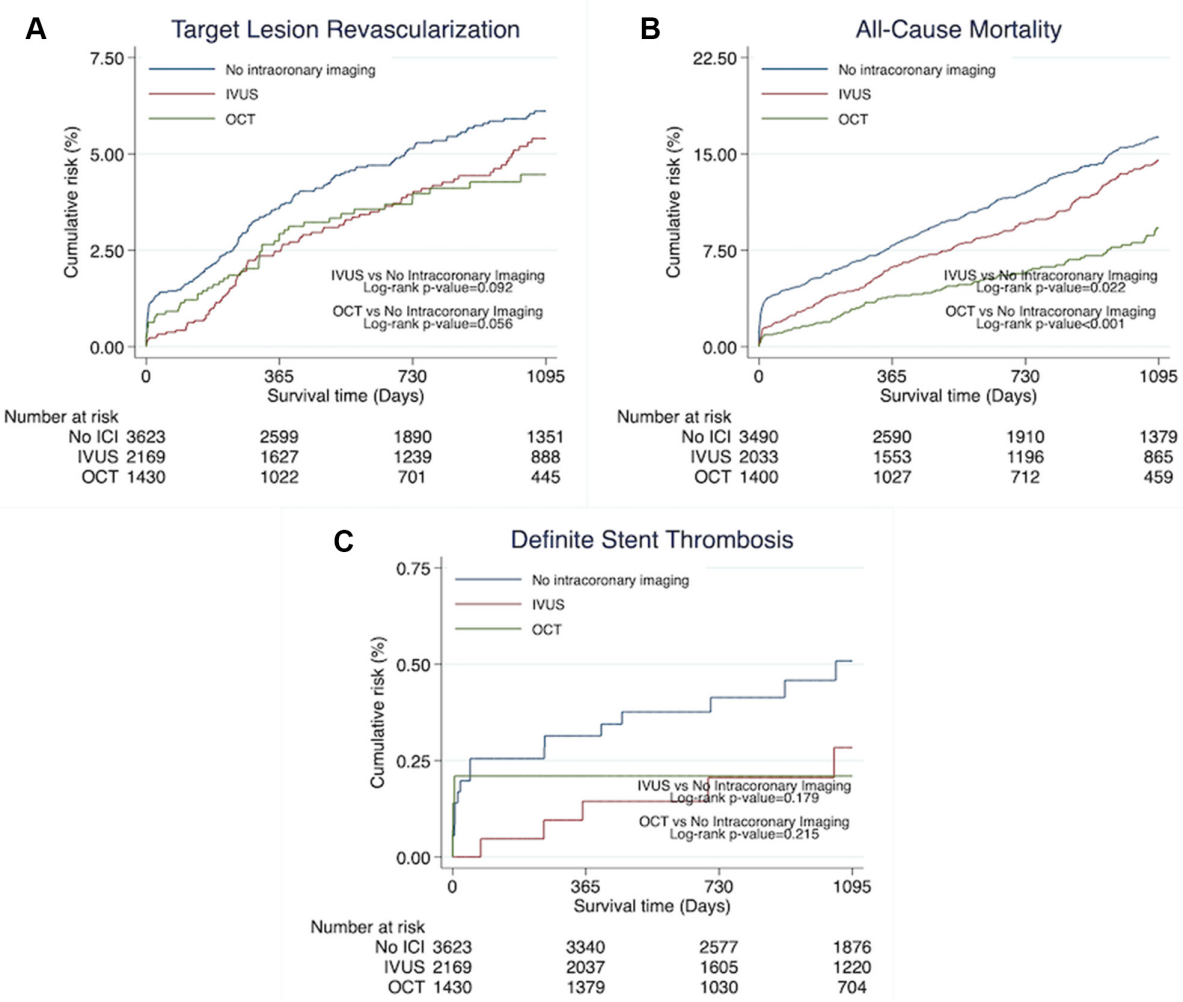
**IVUS and OCT comparison.** Time-to-event Kaplan-Meier curves illustrating the event rate of target lesion revascularization (**A**), all-cause mortality (**B**), and definite stent thrombosis (**C**) comparing event rates between IVUS, OCT, and no intracoronary imaging. Twenty-four lesions were excluded from this analysis because they had undergone both IVUS and OCT. ICI, intracoronary imaging; IVUS, intravascular ultrasound; OCT, optical coherence tomography.

Intracoronary imaging was associated with significantly lower risk of the primary end point, TLR (3.7% vs 4.7%; HR, 0.77; 95% CI, 0.61-0.97; *P* = .025), as well as significantly lower risk of all-cause mortality (9.1% vs 12.8%; HR, 0.70; 95% CI, 0.61-0.81; *P* < .001) (Figure 2 and Table 2). There were no differences in definite stent thrombosis (0.2% vs 0.4%; HR, 0.49; 95% CI, 0.21-1.16; *P* = .106). The SCAAR registry does not capture probable stent thrombosis. However, a post hoc sensitivity analysis was carried out to simulate this. Within the first 30 days, TLR and/or death occurred in 236 patients. Assuming all deaths were due to fatal myocardial infarction because of not reaching catheter laboratory in time, TLR or death during the first 30 days was used as a surrogate variable for probable stent thrombosis. Using this definition, we observed a significant reduction of probable stent thrombosis when intracoronary imaging was used (1.8% vs 5.0%; HR, 0.3; 95% CI, 0.27-0.47; *P* < .001). For the LMCA, intracoronary imaging was associated with significantly lower rates of TLR (HR, 0.66; 95% CI, 0.49-0.89; *P* = .006) and all-cause mortality (HR, 0.62; 95% CI, 0.52-0.72; *P* < .001) (Figure 3). A significant intracoronary imaging-by-subgroup interaction was observed with high-volume intracoronary imaging centers associated with a significantly lower risk of TLR compared with low-volume intracoronary imaging centers (interaction *P* value, .021) (Figure 4), and the IVUS subgroup analysis was in line with this result (interaction *P* value, .038) (Figure 4) but not OCT. However, LMCA lesions were observed as an effect modifier for OCT showing a significant reduction in TLR when OCT was used for LMCA lesions compared with non-LMCA lesions (interaction *P* value, .013) (Figure 4). When analyzing IVUS and OCT independently, the results were consistent with the main analysis, showing lower rates of TLR and all-cause mortality when IVUS or OCT was used (Figure 5 and Supplementary Table 1).

**Table 2:** Results.

End point	Intracoronary imaging	No intracoronary imaging	HR (95% CI)	*P*
Target lesion revascularization	134/3623 (3.7%)	170/3623 (4.7%)	0.77 (0.61-0.97)	.025
All-cause mortality	315/3457 (9.1%)	448/3490 (12.8%)	0.70 (0.61-0.81)	<.001
Definite stent thrombosis	8/3623 (0.2%)	16/3623 (0.4%)	0.49 (0.21-1.16)	.106

HR, hazard ratio.

## Discussion

In this nationwide analysis on proximal coronary artery lesions contemporarily treated with drug-eluting stents, the use of intracoronary imaging compared to angiography alone was associated with fewer repeat revascularizations and lower risk of all-cause mortality. The benefit of intracoronary imaging was primarily driven by improved outcome after revascularization of LMCA lesions. In-depth analysis revealed different effect modifiers for IVUS and OCT separately. Use of IVUS was an effect modifier, with high-volume intracoronary imaging centers showing a lower risk of repeat revascularization and mortality, whereas OCT in LMCA lesions was observed to be an effect modifier associated with improved outcome (Central Illustration).

**Central Illustration:**
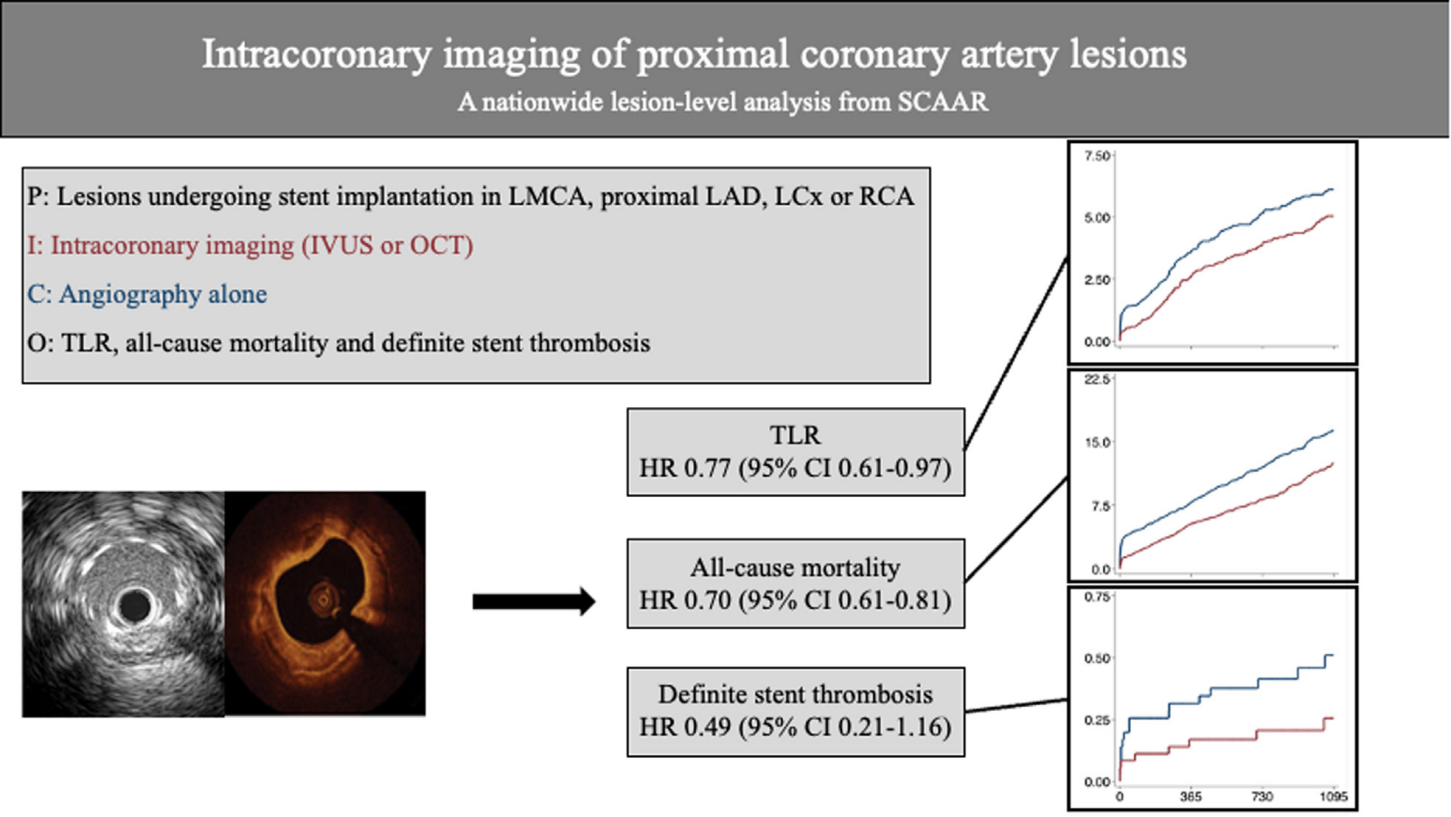
HR, hazard ratio; IVUS, intravascular ultrasound; LAD, left anterior descending; LCx, left circumflex artery; LMCA, left main coronary artery; OCT, optical coherence tomography; PCI, percutaneous coronary intervention; RCA, right coronary artery; SCAAR, Swedish Coronary Angiography and Angioplasty Registry; TLR, target lesion revascularization.

Several studies have assessed the impact of intracoronary imaging on outcome after PCI. From 2010 to date, we found 11 randomized trials, 33 observational studies, and 17 meta-analyses assessing the impact of intracoronary imaging on outcome (Supplementary Table 4). Our results are in line with previous studies showing an association between intracoronary imaging and lower rates of mortality^5^^,^^7^^,^^9^^,^^13^, ^14^, ^15^, ^16^ and lower rates of TLR,^3^^,^^5^^,^^17^, ^18^, ^19^, ^20^ and both lower mortality and TLR in complex lesions.^21^ However, it should be acknowledged that data on mortality reductions come primarily from observational data.^13^, ^14^, ^15^, ^16^ Despite fewer studies, use of OCT seems to be on par with IVUS in lowering cardiovascular events and in line with our study.^9^^,^^22^^,^^23^ Although there is strong support for routine intracoronary imaging when treating LMCA lesions with studies showing both reduced mortality^13^, ^14^, ^15^^,^^24^ and TLR,^20^ the role of intracoronary imaging guiding PCI in other proximal lesions is less established. Even though it is conceivable that imaging improves stent sizing and apposition beyond the LMCA, the LMCA remains a larger vessel that is more difficult to size, and the additive information of intracoronary imaging here may be of greater importance for the more technically challenging procedures.^25^ Our results did not indicate lesion location to be an effect modifier in the interaction tests, suggesting that the benefits of intracoronary imaging might not only be limited to LMCA lesions. However, the LMCA constituted nearly half of all lesions with an associated relative risk reduction of 35% compared to 5% for non-LMCA lesions. This is in line with a previous study based on SCAAR data, showing an association between IVUS and lower rate of all-cause mortality for patients with LMCA lesions.^13^ Differences between this study include the assessment of all proximal coronary arteries; larger study population, allowing better power for this assessment; and the use of OCT.

Previous studies have also shown that intracoronary imaging reduces the rate of stent thrombosis.^5^^,^^17^ We observed a 50% lower relative risk of definite stent thrombosis that was not observed to be statistically significant due to few events resulting in wide confidence intervals. The strict definition of angiographically verified stent thrombosis most likely led to an underestimation of the true event rate of stent thrombosis. However, stent thrombosis may have contributed to the difference in mortality as stent thrombosis in a proximal coronary artery is often fatal, and the patient might not reach the PCI laboratory in time for diagnosis. As the SCAAR registry only collects information on patients undergoing coronary angiography, target vessel myocardial infarction without angiographic confirmation is not available, rendering us unable to estimate rates of probable stent thrombosis. However, we tried to emulate this analysis by assuming that all deaths were due to stent thrombosis. Under this assumption, intracoronary imaging was associated with a significant reduction in probable stent thrombosis. These results should be interpreted carefully as the results are based on a surrogate variable for probable stent thrombosis.

In-depth analyses of various subgroups revealed that intracoronary imaging performed in high-volume PCI centers was associated with lower risk of TLR compared with intracoronary imaging used in low-volume centers. A similar association was made for IVUS separately but not for OCT. Instead, the LMCA was observed as effect modifier for use of OCT, with OCT used in the LMCA being associated with a potent reduction in TLR compared to when OCT was used in non-LMCA lesions. More frequent use of intracoronary imaging provides experience and improves interpretation of results. Experienced PCI operators have been linked to better outcome after revascularization of complex lesions compared to less experienced PCI operators, and thus, it is conceivable that in the hands of more experienced operators, intracoronary imaging seems to convey an even larger benefit.^26^

Finally, despite current evidence regarding the benefit of intracoronary imaging, only 5.8% of all proximal coronary lesions prior to PS matching were assessed with IVUS/OCT in this real-world setting. This rate is lower than what is observed in the recently published Fractional Flow Reserve-Guided PCI as Compared with Coronary Bypass Surgery (FAME-3) in which IVUS/OCT was utilized in 12%.^27^ However, whether intracoronary imaging should be used as a routine strategy or per operator decision needs to be studied further. The fact that routine intracoronary imaging is not indispensable is supported by the study by Kim et al,^28^ which randomized patients to IVUS or no IVUS, showing that the use of IVUS was associated with improved outcome when it was used per operator decision but not as a routine strategy.

### Limitations

This study has several limitations. A selection bias is possible due to the low usage of intracoronary imaging. To address selection bias and confounding, we performed PS matching based on variables associated with the tendency to have intracoronary imaging performed. These variables were carefully selected a priori based on previous literature and clinical experience. Most baseline differences between groups were neutralized after PS matching. However, a few differences remained such as larger stent size or fewer and shorter stents deployed when intracoronary imaging was used. These differences are the result of intracoronary imaging usage and mediators of outcome and not indicative of underlying confounding and as such, should not be adjusted for. The Kaplan-Meier curves for all-cause mortality separate immediately, possibly due to early stent thrombosis or in-stent restenosis as stent-related events are known to happen early. However, underlying confounding cannot be ruled out due to the observational nature of this study. More specific limitations are related to how information is collected in the SCAAR registry. Although the registry introduced a new diagnostic module allowing for better data capture when intracoronary diagnostic modalities are performed, certain variables are not mandatory, and as a result, IVUS/OCT measurements of vessel diameter or minimum stent area are not available.

## Conclusion

The use of intracoronary imaging in proximal coronary artery lesions is associated with lower rates of repeat revascularization and better survival. Results appear to be primarily driven by improved outcome of LMCA lesions. These results reinforce the importance of using intracoronary imaging in proximal coronary arteries.

## Declaration of competing interest

The author(s) declared no potential conflicts of interest with respect to the research, authorship, and/or publication of this article.

## Figures and Tables

**Supplementary Figure 1:**