# Supplementary material for: Intracoronary Imaging of Proximal Coronary Artery Lesions – A Nationwide Lesion-Level Analysis From SCAAR
Source: J Soc Cardiovasc Angiogr Interv. 2023 Feb 20;2(3):100597. doi: 10.1016/j.jscai.2023.100597 (PMC11307536; doi:10.1016/j.jscai.2023.100597)
Supplement: Supplementary Data [file mmc1.docx]

Supplementary material

**Intracoronary imaging of proximal coronary artery lesions – a nationwide lesion-level analysis from SCAAR**

Sacharias von Koch B.Med.Sc ^1^, Sofia Bergman, MD^1^, Pontus Andell MD, PhD ^2^, Göran K Olivecrona MD, PhD^1^, Matthias Götberg MD, PhD^1^, Elmir Omerovic Prof ^3^, Ole Fröbert, Prof ^4^, Sergio Buccheri MD^5^, Stefan James Prof^5^, Sasha Koul MD, PhD^1^, Moman A. Mohammad, MD, PhD^1^ and David Erlinge, Prof^1,6^

1. Department of Cardiology, Clinical Sciences, Lund University, Skåne University Hospital, Lund, Sweden
2. Heart and Vascular Theme, Karolinska University Hospital, and Unit of Cardiology, Department of Medicine, Karolinska Institutet, Stockholm, Sweden
3. Department of Cardiology Sahlgrenska University Hospital Gothenburg Sweden
4. Örebro University, Faculty of Health, Department of Cardiology, Örebro, Sweden and Department of Clinical Medicine, Aarhus University Health, Aarhus, Denmark.
5. Department of Medical Sciences, Uppsala University, and Uppsala Clinical Research Center, Uppsala, Sweden
6. Division of Cardiology, University of California San Diego, San Diego, USA

**Corresponding author**

Prof David Erlinge, Department of Cardiology, Clinical Sciences, Lund University, Lund, Sweden

Phone: +46733746165

E-mail: david.erlinge@med.lu.se/David.erlinge@gmail.com

#

Table of content:

[**Supplementary table 1.** IVUS and OCT comparison. 3](#_Toc125233035)

[**Supplementary table 2.** Study population characteristics before PS-matching. 4](#_Toc125233036)

[**Supplementary table 3.** Mean propensity score before and after matching. 7](#_Toc125233037)

[**Supplementary table 4.** Previous research. 8](#_Toc125233038)

[**Supplementary table 5.** Different follow-up times for each endpoint. 14](#_Toc125233039)

[**Supplementary table 6.** Study population characteristics after converting to patient-level. 15](#_Toc125233040)

[**Supplementary table 7.** Sensitivity analysis of all-cause mortality. 17](#_Toc125233041)

[**Supplementary figure 1.** Standardized mean differences from propensity score matching. 18](#_Toc125233042)

[**References** 19](#_Toc125233043)

# **Supplementary table 1.** IVUS and OCT comparison.

| Endpoint | Population | IVUS | OCT | No intracoronary imaging |
| --- | --- | --- | --- | --- |
| Target lesion revascularization | Total study population (n=7222) | 85/2169 (3.9%) | 49/1430 (3.4%) | 170/3623 (4.7%) |
| All-cause mortality | Total study population (n=6923) | 224/2033 (11.0%) | 87/1400 (6.2%) | 448/3490 (12.8%) |
| Definite stent thrombosis | Total study population (n=7222) | 5/2169 (0.2%) | 3/1430 (0.2%) | 16/3623 (0.4%) |

# **Supplementary table 2.** Study population characteristics before PS-matching.

| Patient characteristics | | Intracoronary imaging (n=4158) | No intracoronary imaging (n=67414) | P value | Missing |
| --- | --- | --- | --- | --- | --- |
| Demographics | | | | |  |
| Age, y | | 68.2 (67.8-68.5) | 69.1 (69.0-69.2) | <0.001 | 0.0 |
| Age >75 | | 1128 (27.1) | 20146 (29.9) | <0.001 | 0.0 |
| Male | | 3255 (78.3) | 49812 (73.9) | <0.001 | 0.0 |
| Current smoker | | 582 (14.8) | 11325 (17.8) | <0.001 | 5.6 |
| Comorbidities | | | | |  |
| Diabetes mellitus | | 873 (21.1) | 15020 (22.4) | 0.045 | 0.7 |
| Hypertension | | 2811 (68.1) | 43651 (65.5) | 0.001 | 1.1 |
| Previous MI^a^ | | 1023 (24.9) | 15676 (23.7) | 0.086 | 1.8 |
| Previous PCI^b^ | | 1114 (26.8) | 15147 (22.5) | <0.001 | 0.0 |
| Previous CABG^c^ | | 264 (6.4) | 4671 (6.9) | 0.151 | 0.0 |
| Hyperlipidemia | | 2483 (60.1) | 34349 (51.7) | <0.001 | 1.4 |
| Estimated GFR^d^ | | 80.8 (80.0-81.7) | 81.0 (80.7-81.2) | 0.776 | 25.6 |
| Chronic kidney disease stage | Stage I–II | 2885 (82.4) | 40848 (82.2) | 0.069 | 25.7 |
|  | Stage III | 533 (15.2) | 7889 (15.9) |  |  |
|  | Stage IV–V | 84 (2.4) | 938 (1.9) |  |  |
| In-hospital characteristics | | | | |  |
| Inclusion time | Early (2013–2015) | 1032 (24.8) | 25340 (37.6) | <0.001 | 0.0 |
|  | Mid (2016–2018) | 2112 (50.8) | 30905 (45.8) |  |  |
|  | Late (2019–2021) | 1014 (24.4) | 11169 (16.6) |  |  |
| High-volume PCI^b^ center | | 2897 (69.7) | 34839 (51.7) | <0.001 | 0.0 |
| High-volume intracoronary imaging center | | 3329 (80.1) | 35423 (52.6) | <0.001 | 0.0 |
| Indication | Stable CAD^e^ | 1253 (30.1) | 15073 (22.4) | <0.001 | 0.0 |
|  | Unstable angina | 700 (16.8) | 10291 (15.3) |  |  |
|  | NSTEMI^f^ | 1379 (33.2) | 20704 (30.7) |  |  |
|  | STEMI^g^ | 487 (11.7) | 17172 (25.5) |  |  |
|  | Other | 339 (8.2) | 4174 (6.2) |  |  |
| Urgency | Elective | 1487 (37.3) | 18602 (28.8) | <0.001 | 4.2 |
|  | Subacute | 1878 (47.2) | 26223 (40.6) |  |  |
|  | Acute | 617 (15.5) | 19735 (30.6) |  |  |
| Killip class at presentation | 1 | 3537 (96.5) | 54936 (96.5) | 0.869 | 15.3 |
|  | 2 | 127 (3.5) | 2003 (3.5) |  |  |
| Medical treatment prior to PCI or periprocedural added medical treatment | | | | | |
| Dual antiplatelet therapy | | 4179 (96.5) | 63840 (95.9) | 0.028 | 0.1 |
| Aspirin | | 4249 (98.1) | 64845 (97.3) | 0.003 | 0.1 |
| Clopidogrel | | 1543 (35.6) | 18813 (28.2) | <0.001 | 0.1 |
| Prasugrel | | 27 (0.6) | 696 (1.0) | 0.007 | 0.0 |
| Ticagrelor | | 2763 (63.8) | 46994 (70.5) | <0.001 | 0.1 |
| Heparin | | 4042 (93.2) | 62956 (94.5) | 0.001 | 0.0 |
| Bivalirudin | | 270 (6.2) | 7089 (10.6) | <0.001 | 0.0 |
| Fondaparinux | | 906 (20.9) | 14685 (22.0) | 0.079 | 0.1 |
| GP^j^ IIb/IIIa inhibitor | | 183 (4.2) | 2578 (3.9) | 0.245 | 0.0 |
| Procedure characteristics | | | | |  |
| Concomitantly stented segments | 1 | 2683 (64.5) | 53100 (78.8) | <0.001 | 0.0 |
|  | 2 | 1182 (28.4) | 12138 (18.0) |  |  |
|  | 3 | 274 (6.6) | 1991 (3.0) |  |  |
|  | 4 | 19 (0.5) | 185 (0.3) |  |  |
| Vascular approach | A. Femoralis | 868 (20.9) | 10854 (16.1) | <0.001 | 0.1 |
|  | A. Radialis | 3168 (76.3) | 55096 (81.8) |  |  |
|  | Other | 115 (2.8) | 1397 (2.1) |  |  |
| Fluoroscopy time, min | | 25.3 (24.8-25.8) | 19.2 (19.1-19.3) | <0.001 | 0.0 |
| Contrast volume, mL | | 208.2 (205.5-211.0) | 170.6 (170.0-171.2) | <0.001 | 0.0 |
| Number of stents | 1 | 1485 (35.7) | 26568 (39.4) | <0.001 | 0.0 |
|  | 2 | 1193 (28.7) | 20334 (30.2) |  |  |
|  | 3 or more | 1480 (35.6) | 20512 (30.4) |  |  |
| Aortic balloon pump | | 17 (0.4) | 97 (0.1) | <0.001 | 0.0 |
| Segment characteristics | | | | |  |
| Segments stented | LMCA^k^ | 2109 (50.7) | 4345 (6.5) | <0.001 | 0.0 |
|  | Proximal LAD^l^ | 1559 (37.5) | 33614 (49.9) |  |  |
|  | Proximal LCx^m^ | 255 (6.1) | 13679 (20.3) |  |  |
|  | Proximal RCA^n^ | 235 (5.7) | 15776 (23.4) |  |  |
| ACC/AHA^o^ lesion classification | Type A | 143 (3.5) | 4768 (7.1) | <0.001 | 0.1 |
|  | Type B1–B2 | 1502 (3.6) | 38906 (57.8) |  |  |
|  | Type C or B1–B2 with bifurcation | 2504 (60.4) | 23686 (35.2) |  |  |
| Thrombus aspiration | | 78 (1.9) | 1955 (2.9) | <0.001 | 0.0 |
| Direct stent vs balloon and stent | Direct stent | 682 (16.4) | 9988 (14.8) | 0.005 | 0.0 |
|  | Balloon and stent | 3476 (83.6) | 57426 (85.2) |  |  |
| Drug-eluting stent | | 4125 (99.5) | 66131 (98.4) | <0.001 | 0.3 |
| Stent length, mm | | 23.1 (22.8-23.4) | 22.6 (22.6-22.7) | <0.001 | 0.0 |
| Stent diameter, mm | | 4.0 (4.0-4.0) | 3.3 (3.3-3.3) | <0.001 | 0.0 |
| Stent diameter categories | <3.00 | 128 (3.1) | 13324 (19.8) | <0.001 | 0.0 |
|  | 3.00 to <3.50 | 440 (10.6) | 21633 (32.1) |  |  |
|  | 3.50 to <4.00 | 1066 (25.6) | 22262 (33.0) |  |  |
|  | 4.00 to <4.50 | 1110 (26.7) | 7632 (11.3) |  |  |
|  | >4.50 | 1414 (34.0) | 2555 (3.8) |  |  |
| Max pressure in balloon (atm) | | 19.5 (19.4-19.6) | 19.0 (19.0-19.0) | <0.001 | 0.3 |
| Post dilatation | | 3470 (83.5) | 31337 (46.5) | <0.001 | 0.0 |

^a^ Myocardial infarction

^b^ Percutaneous coronary intervention

^c^ Coronary artery bypass grafting

^d^ Glomerular filtration rate

^e^ Chronic coronary syndrome

^f^ Non-ST elevated myocardial infarction

^g^ ST elevated myocardial infarction

^h^ Dual antiplatelet therapy

^i^ Low molecular weight heparin

^j^ Glycoprotein

^k^ Left main coronary artery

^l^ Left anterior descending artery

^m^ Left circumflex artery

^n^ Right coronary artery

^o^ The American College of Cardiology/American Heart Association classification

# **Supplementary table 3.** Mean propensity score before and after matching.

|  | Intracoronary imaging | No intracoronary imaging | P-value |
| --- | --- | --- | --- |
| Before PS-matching | 0.25 | 0.05 | <0.001 |
| After PS-matching | 0.22 | 0.22 | 0.412 |

# **Supplementary table 4.** Previous research.

| **Author** | **Publication year** | **Number of patients** | **Left main only** | **IVUS or OCT** | **Study design** | **Follow-up time (months)** | **TLR (%) ICI vs no ICI** | **Death (%) ICI vs no ICI** | **ST (%) ICI vs no ICI** | **Other** | |
| --- | --- | --- | --- | --- | --- | --- | --- | --- | --- | --- | --- |
| Jakabcin et al^1^ | 2010 | 210 | No | IVUS | RCT | 18 | 6 vs 6, P=NS | 3 vs 2, P=NS | 4 vs 6, P=NS | Stent thrombosis definition: Definite/probable/possible Death definition: All-cause | |
| Kim et al^2^ | 2010 | 758 | No | IVUS | Observational study | 48 | NR | 0.4 vs 3.6, P=0.001 | 0.7 vs 0.9, P=0.79 | 65% DES only Stent thrombosis definition: Definite/probable/possible Death definition: All-cause | |
| Kim et al^3^ | 2011 | 1668 | No | IVUS | Observational study | 36 | 7.4 vs 6.7, P=0.76 | 3.1 vs 3.6, P=0.29 | 0.2 vs 0.6, P=0.29 | Stent thrombosis definition: Definite/probable/possible Death definition: All-cause | |
| Claessen et al^4^ | 2011 | 1504 | No | IVUS | Observational study | 24 | NR | 3.1 vs 3.8, P=0.39 | 0.6 vs 1.0, P=0.48 | Stent thrombosis definition: Definite/probable Death definition: All-cause | |
| Zhang et al^5^ | 2012 | 19619 | No | IVUS | Meta-analysis | 6-48 | 0.90 (0.73-1.11) | 0.59 (0.48-0.73) | 0.58 (0.44-0.77) | Stent thrombosis definition: Definite/probable Death definition: All-cause and cardiovascular | |
| Wakabayashi et al^6^ | 2012 | 1984 | No | IVUS | Observational study | 12 | 9.2 vs 10.6, P=0.42 | 5.9 vs 8.4, P=0.077 | 0.9 vs 0.6, P=0.53 | Stent thrombosis definition: Definite Death definition: All-cause | |
| Prati et al^7^ | 2012 | 670 | No | OCT | Observational study | 12 | 3.3 vs 3.3, P=1.0 | 3.3 vs 6.9, P=0.035 | 0.3 vs 0.6, P=1.0 | Stent thrombosis definition: Definite Death definition: All-cause | |
| Kim et al^8^ | 2013 | 543 | No | IVUS | RCT | 12 | NR | 1.1 vs 0.7, P=0.64 | 0.4 vs 0.4, P=1.00 | In the intention to treat analysis Stent thrombosis definition: Definite/probable/possible Death definition: All-cause | |
| Yoon et al^9^ | 2013 | 1574 | No | IVUS | Observational study | 12 | NR | 0.2 vs 0.8, P=0.122 | 0.2 vs 0.2, P=0.754 | Stent thrombosis definition: Not defined Death definition: All-cause | |
| Hur et al^10^ | 2013 | 8371 | No | IVUS | Observational study | 36 | NR | 2.7 vs 5.4, P<0.001 | 1.8 vs 2.1, P=0.613 | In the DES-population Stent thrombosis definition: Definite/probable/possible Death definition: All-cause | |
| Chieffo et al^11^ | 2013 | 284 | No | IVUS | RCT | 24 | 9.2 vs 11.9, P=NS | 0 vs 1.4, P=NS | P=NS | Only one reported definite stent thrombosis Stent thrombosis definition: Definite Death definition: Cardiovascular | |
| Klersy et al^12^ | 2013 | 18707 | No | IVUS | Meta-analysis | 6-18 | NR | 0.60 (0.48-0.74) | 0.50 (0.32-0.80) | Stent thrombosis definition: Not defined Death definition: All-cause and cardiovascular | |
| Chen et al^13^ | 2013 | 628 | No | IVUS | Observational study | 12 | 10.6 vs 11.4, P=1.000 | 0 vs 4.1, P=0.060 | 0.8 vs 7.3, P=0.019 | Stent thrombosis definition: Definite Death definition: All-cause | |
| Park et al^14^ | 2013 | 1421 | No | IVUS | Observational study | 12 | 1.1 vs 0.6, P=0.484 | 1.9 vs 1.7, P=0.442 | 0.2 vs 0.2, P=1.000 | Stent thrombosis definition: Definite/probable Death definition: All-cause | |
| Witzenbichler et al^15^ | 2014 | 8583 | No | IVUS | Observational study | 12 | 1.5 vs 2.4, P=0.007 | 1.8 vs 2.0, P=0.40 | 0.6 vs 1.0, P=0.02 | Stent thrombosis definition: Definite/probable Death definition: All-cause | |
| Ahn et al^16^ | 2014 | 26503 | No | IVUS | Meta-analysis | 12-48 | 0.81 (0.66-1.00) | 0.61 (0.48-0.79) | 0.59 (0.47-0.75) | Stent thrombosis definition: Not defined Death definition: Not defined | |
| Hong et al^17^ | 2014 | 2568 | No | IVUS | Observational study | 24 | 10.4 vs 11.9, P=0.631 | 2.0 vs 3.0, P=0.527 | 0 vs 3.0, P=0.014 | Stent thrombosis definition: Definite/probable Death definition: All-cause | |
| Jang et al^18^ | 2014 | 24849 | No | IVUS | Meta-analysis | NA | NR | 2.3 vs 3.3, P<0.001 | 1.1 vs 1.7, P=0.002 | Stent thrombosis definition: Definite/probable Death definition: All-cause | |
| de la Torre Hernandez et al^19^ | 2014 | 1670 | Yes | IVUS | Observational study | 36 | 7.7 vs 6.3, P=0.7 | 7.4 vs 13.0, P=0.01 | 0.6 vs 2.2, P=0.04 | Stent thrombosis definition: Definite/probable Death definition: All-cause | |
| Gao et al^20^ | 2014 | 1016 | Yes | IVUS | Observational study | 12 | 2.4 vs 9.4, P<0.001 | 1.8 vs 6.2, P=0.002 | 0 vs 1.3, P=0.077 | Stent thrombosis definition: Definite Death definition: Cardiovascular | |
| Hong et al^21^ | 2015 | 1400 | No | IVUS | RCT | 12 | 2.5 vs 5.0, P=0.02 | 0.4 vs 0.7, P=0.48 | 0.3 vs 0.3, P>0.99 | Stent thrombosis definition: Definite/probable Death definition: Cardiovascular | |
| Tian et al^22^ | 2015 | 230 | No | IVUS | RCT | 24 | 7.6 vs 10.4, P=0.484 | 5.2 vs 6.1, P=0.775 | 0.9 vs 5.2, P=0.052 | Stent thrombosis definition: Definite Death definition: All-cause | |
| Kim et al^23^ | 2015 | 402 | No | IVUS | RCT | 12 | 2.6 vs 4.1, P=0.4 | 1.0 vs 1.5, P=0.66 | 0 vs 1.5, P=0.11 | Stent thrombosis definition: Definite/probable Death definition: All-cause | |
| Zhang et al^24^ | 2015 | 29068 | No | IVUS | Meta-analysis | 12-48 | 0.92 (0.76-1.11) | 0.62 (0.54-0.71) | 0.59 (0.47-0.73) | Stent thrombosis definition: Definite/probable Death definition: All-cause and cardiovascular | |
| Wijns et al^25^ | 2015 | 418 | No | OCT | Observational study | 1 | 1.5 vs 0, P=0.485 | 0 vs 0.7, P=0.596 | 0 vs 0, P=1.000 | This study has four groups: 1. No OCT 2.  OCT before PCI 3. OCT after PCI 4.  OCT before and after PCI. 4 vs 1 is presented here.  Stent thrombosis definition: Definite Death definition: All-cause | |
| Tan et al^26^ | 2015 | 123 | Yes | IVUS | RCT | 24 | 8.2 vs 19.4, P=0.045 | 3.3 vs 4.8, P=0.648 | NR | Death definition: Cardiovascular | |
| Elgendy et al^27^ | 2016 | 3192 | No | IVUS | Meta-analysis | 12-24 | 0.60 (0.43-0.84) | 0.60 (0.21-1.00) | 0.49 (0.24-0.99) | Stent thrombosis definition: Definite/probable Death definition: Cardiovascular | |
| Zhang et al^28^ | 2016 | 84 | No | IVUS | RCT | 12 | NR | 0 vs 0, P=NS | NR | Death definition: Cardiovascular | |
| Patel et al^29^ | 2016 | 225 | No | IVUS | Observational study | 50.4 | 7 vs 25, P=0.002 | 9 vs 8, P=1.00 | 0 vs 1, P=1.00 | Stent thrombosis definition: Not defined Death definition: Cardivascular | |
| Nakatsuma et al^30^ | 2016 | 3028 | No | IVUS | Observational study | 60 | NR | 13 vs 17, P=0.77 | 2.2 vs 1.3, P=0.41 | Results from the DES analysis Stent thrombosis definition: Definite Death definition: All-cause | |
| Shin et al^31^ | 2016 | 2345 | No | IVUS | Meta-analysis | 12 | 0.61 (0.40-0.93) | 0.38 (0.10-1.42) | 0.50 (0.13-2.01) | Stent thrombosis definition: Definite/probable/possible Death definition: Cardiovascular | |
| Steinvil et al^32^ | 2016 | 31283 | No | IVUS | Meta-analysis | 9-48 | 0.77 (0.67-0.89) | 0.62 (0.54-0.72) | 0.58 (0.47-0.73) | Stent thrombosis definition: Definite/probable Death definition: All-cause and cardiovascular | |
| Ali et al^33^ | 2016 | 450 | No | OCT | RCT | 1 | 1 vs 1, P=NS | 0 vs 0, P=NS | 1 vs 0, P=NS | Stent thrombosis definition: Not defined Death definition: All-cause | |
| Meneveau et al^34^ | 2016 | 240 | No | OCT | RCT | 6 | 0 vs 0, P=NS | 1 vs 0, P=NS | 0 vs 0, P=NS | Stent thrombosis definition: Not defined Death definition: All-cause | |
| Sheth et al^35^ | 2016 | 10732 | No | OCT | Observational study | 12 | NR | 1.9 vs 3.7, P=0.2 | 1.9 vs 1.2, P=0.49 | Stent thrombosis definition: Not defined Death definition: Cardivascular | |
| Bavishi et al^36^ | 2017 | 3276 | No | IVUS | Meta-analysis | 12-24 | 0.62 (0.45-0.86) | 1.00 (0.48-2.09) | 0.57 (0.26-1.23) | Stent thrombosis definition: Definite/probable Death definition: All-cause | |
| Qian et al^37^ | 2017 | 3192 | No | IVUS | Meta-analysis | 12-24 | 0.60 (0.42-0.85) | 0.47 (0.19-1.15) | 0.56 (0.25-1.23) | Stent thrombosis definition: Definite/probable Death definition: Cardiovascular | |
| Buccheri et al^38^ | 2017 | 17882 | No | IVUS | Meta-analysis | 1-36 | 0.74 (0.58-0.90) | 0.74 (0.58-0.98) | 0.42 (0.20-0.72) | Stent thrombosis definition: Not defined Death definition: All-cause | |
| Nerlekar et al^39^ | 2017 | 9313 | No | IVUS | Meta-analysis | NA | 0.61 (0.42-0.90) | 0.33 (0.14-0.78) | 0.31 (0.12-0.78) | Stent thrombosis definition: Definite/probable Death definition: All-cause | |
| Iannaccone et al^40^ | 2017 | 1832 | No | OCT | Observational study | 23 | 2 vs 3, P=0.92 | 3 vs 4, P=0.15 | 0 vs 2.7, 0.26 | Stent thrombosis definition: Not defined Death definition: All-cause | |
| Andell et al^41^ | 2017 | 2468 | Yes | IVUS | Observational study | 120 | NR | 10.9 vs 18.5, P=0.003 | 0 vs 0.3, P=NA | Stent thrombosis definition: Definite Death definition: All-cause | |
| Kim et al^42^ | 2017 | 196 | Yes | IVUS | Observational study | 36 | 20 vs 6, P=0.346 | 7 vs 22, P=0.124 | NR | Death definition: All-cause | |
| Tian et al^43^ | 2017 | 1899 | Yes | IVUS | Observational study | 36 | 3.1 vs 3.3, P=0.64 | 3.8 vs 2.2, P=0.007 | 1.4 vs 1.7, P=0.37 | All-cause death was not significant in the  unadjusted analysis Stent thrombosis definition: Definite/probable Death definition: All-cause | |
| Ye et al^44^ | 2017 | 6480 | Yes | IVUS | Meta-analysis | 12-120 | 0.43 (0.25-0.73) | 0.60 (0.47-0.75) | 0.28 (0.12-0.67) | Stent thrombosis definition: Definite/probable Death definition: All-cause | |
| Zhang et al^45^ | 2018 | 1448 | No | IVUS | RCT | 12 | 1.2 vs 2.6, P=0.05 | 0.7 vs 1.4, P=0.19 | 0 vs 0.3, P=0.16 | Results from the lesion-level analysis Stent thrombosis definition: Definite Death definition: Cardiovascular | |
| Maehara et al^46^ | 2018 | 8583 | No | IVUS | Observational study | 24 | 5.0 vs 6.5, P=0.01 | 3.3 vs 4.2, P=0.03 | 0.46 vs 0.85, P=0.04 | Stent thrombosis definition: Definite Death definition: All-cause | |
| Smilowitz et al^47^ | 2018 | 3211872 | No | IVUS and OCT | Observational study | In hospital | NR | 0.9 vs 1.8, P<0.001 | NR | Death definition: All-cause | |
| Wang et al^48^ | 2018 | 4592 | Yes | IVUS | Meta-analysis | 12-36 | 0.60 (0.31-1.18) | 0.55 (0.42-0.71) | 0.48 (0.27-0.84) | Stent thrombosis definition: Definite/probable/possible Death definition: All-cause | |
| Choi et al^49^ | 2019 | 6005 | No | IVUS | Observational study | 64 | 8.3 vs 11.4, P=0.008 | 17.1 vs 25.5, P<0.001 | 3.1 vs 4.4, P=0.008 | Complex lesions Stent thrombosis definition: Definite/probable Death definition: All-cause | |
| Kim et al^50^ | 2020 | 11731 | No | IVUS | Observational study | 12 | 1.7 vs 1.5, P=0.597 | 4.4 vs 7.0, P<0.001 | NR | Death definition: All-cause | |
| Darmoch et al^51^ | 2020 | 27610 | No | IVUS | Meta-analysis | 6-64 | 0.81 (0.70-0.94 | 0.63 (0.54-0.73) | 0.57 (0.41-0.79) | Stent thrombosis definition: Definite/probable Death definition: All-cause | |
| Hong et al^52^ | 2020 | 1400 | No | IVUS | Observational study | 60 | 4.8 vs 8.4, P=0.007 | 0.9 vs 2.2, P=0.074 | 0.3 vs 0.3, P=1.000 | Stent thrombosis definition: Definite/probable Death definition: Cardiovascular | |
| Iannaccone et al^53^ | 2020 | 69150 | No | IVUS and OCT | Meta-analysis | 12-36 | NR | IVUS: 0.72 (0.52-0.97) OCT: 0.44 (0.25-0.79) | IVUS: 0.34 (0.15-0.90) OCT: 0.08 (0.00-0.62) | Patients from the FFR group are included in  "Number of patients" Stent thrombosis definition: Definite Death definition: All-cause | |
| Park et al^54^ | 2020 | 9525 | No | IVUS | Observational study | 36 | NR | 2.3 vs 3.6, P=0.2 | NR | Death definition: Cardiovascular | |
| Ladwiniec et al^55^ | 2020 | 603 | Yes | IVUS | Observational study | 60 | 5.1 vs 11.6, P=0.01 | 5.5 vs 7.3, P=0.98 | NR | Stent thrombosis definition: Definite/probable Death definition: All-cause | |
| Kinnaird et al^56^ | 2020 | 10112 | Yes | IVUS | Observational study | 12 | NR | 0.66 (0.57-0.77) | NR | Death definition: All-cause | |
| Mentias et al^57^ | 2020 | 207116 | No | IVUS | Observational study | 12 | NR | 11.5 vs 12.3 (p<0.01) | NR | Death definition: All-cause | |
| Gao et al^58^ | 2021 | 1448 | No | IVUS | Observational study | 36 | 5.9 vs 10.2, P=0.003 | 4.3 vs 4.4, P=0.98 | 0.1 vs 1.1, P=0.02 | Stent thrombosis definition: Definite/probable Death definition: All-cause | |
| Hong et al^59^ | 2021 | 1396 | No | IVUS | Meta-analysis | 12 | NR | 0.97 (0.06-15.49) | 1.01 (0.07-17.84) |  | |
| Ali et al^60^ | 2021 | 450 | No | OCT | Observational study | 12 | 1.3 vs 1.4, P=0.99 | 0 vs 0, P=NS | 0.7 vs 0.0, P=0.39 | Stent thrombosis definition: Not defined  Death definition: All-cause | |
| Kang et al^61^ | 2021 | 975 | Yes | IVUS | Observational study | 120 | NR | 16.4 vs 31.0, p<0.001 | NR | Death definition: All-cause | |
|  | | | | | | | | | | |  |

NR, not reported; NS, not significant

# **Supplementary table 5.** Different follow-up times for each endpoint.

| **Time (days)** | **0–30** | **31–365** | **366–1095** |
| --- | --- | --- | --- |
| **TLR, HR (95%CI)** | 0.38 (0.22-0.65), p<0.001 | 0.90 (0.65-1.28), p=0.577 | 0.94 (0.64-1.38), p=0.740 |
| **All-cause mortality, HR (95%CI)** | 0.35 (0.25-0.48), p<0.001 | 0.96 (0.75-1.23), p=0.740 | 0.79 (0.63-0.98), p=0.031 |
| **Definite stent thrombosis, HR (95%CI)** | 0.42 (0.11-1.64), p=0.213 | 0.73 (0.14-3.79), p=0.707 | 0.39 (0.08-2.02), p=0.262 |

TLR, indicates target lesion revascularization; HR, indicates hazard ratio; CI, indicates confidence interval.

# **Supplementary table 6.** Study population characteristics after converting to patient-level.

|  | | Intracoronary imaging (n=3457) | No intracoronary imaging (n=3490) | P-value | Missing |
| --- | --- | --- | --- | --- | --- |
| Demographics | | | | | |
| Age, y | | 68.5 (68.1-68.9) | 68.6 (68.3-69.0) | 0.60 | 0.0 |
| Age >75 | | 967 (28.0) | 948 (27.2) | 0.45 | 0.0 |
| Male | | 2675 (77.4) | 2733 (78.3) | 0.35 | 0.0 |
| Current smoker | | 487 (14.8) | 521 (15.7) | 0.55 | 4.9 |
| Comorbidities | | | | |  |
| Diabetes mellitus | | 753 (21.8) | 739 (21.2) | 0.54 | 0.0 |
| Hypertension | | 2339 (67.7) | 2366 (67.8) | 0.91 | 0.0 |
| Previous MI^a^ | | 852 (24.7) | 899 (25.8) | 0.29 | 0.0 |
| Previous PCI^b^ | | 920 (26.6) | 859 (24.6) | 0.06 | 0.0 |
| Previous CABG^c^ | | 228 (6.6) | 281 (8.1) | 0.02 | 0.0 |
| Hyperlipidemia | | 2040 (59.2) | 1998 (57.5) | 0.15 | 0.3 |
| Estimated GFR^d^ | | 80.6 (79.7-81.5) | 81.0 (80.0-81.9) | 0.56 | 15.7 |
| Chronic kidney disease stage | Stage I–II | 2431 (82.2) | 2383 (82.4) | 0.81 | 15.8 |
|  | Stage III | 455 (15.4) | 435 (15.0) |  |  |
|  | Stage IV–V | 70 (2.4) | 75 (2.6) |  |  |
| In-hospital characteristics | | | | |  |
| Inclusion time | Early (2013–2015) | 935 (27.1) | 1036 (29.7) | 0.04 | 0.0 |
|  | Mid (2016–2018) | 1755 (50.8) | 1685 (48.3) |  |  |
|  | Late (2019–2021) | 767 (22.2) | 769 (22.0) |  |  |
| High-volume intracoronary imaging PCI^b^ center | | 2710 (78.4) | 2773 (79.5) | 0.28 | 0.0 |
| Indication | CCS^e^ | 995 (28.8) | 999 (28.6) | 0.94 | 0.0 |
|  | Unstable angina | 596 (17.2) | 588 (16.9) |  |  |
|  | NSTEMI^f^ | 1174 (34.0) | 1217 (34.9) |  |  |
|  | STEMI^g^ | 428 (12.4) | 429 (12.3) |  |  |
|  | Other | 264 (7.6) | 257 (7.4) |  |  |
| Urgency | Elective | 1253 (36.3) | 1227 (35.2) | 0.49 | 0.0 |
|  | Subacute | 1634 (47.3) | 1699 (48.7) |  |  |
|  | Acute | 570 (16.5) | 564 (16.2) |  |  |
| Killip class at presentation | 1 | 2971 (96.6) | 2915 (96.5) | 0.84 | 12.2 |
|  | 2 | 106 (3.5) | 107 (3.5) |  |  |
| Medical treatment prior to PCI or periprocedural added medical treatment | | | | |  |
| Dual antiplatelet therapy | | 3327 (96.4) | 3337 (95.9) | 0.24 | 0.2 |
| Aspirin | | 3388 (98.1) | 3396 (97.4) | 0.06 | 0.1 |
| Clopidogrel | | 1249 (36.1) | 1228 (35.2) | 0.43 | 0.0 |
| Prasugrel | | 19 (0.6) | 15 (0.4) | 0.48 | 0.0 |
| Ticagrelor | | 2185 (63.3) | 2235 (64.1) | 0.46 | 0.1 |
| Heparin | | 3230 (93.4) | 3235 (92.7) | 0.24 | 0.0 |
| Bivalirudin | | 234 (6.8) | 241 (6.9) | 0.82 | 0.0 |
| Fondaparinux | | 779 (22.5) | 799 (22.9) | 0.72 | 0.0 |
| GP^j^ IIb/IIIa inhibitor | | 146 (4.2) | 117 (3.4) | 0.06 | 0.0 |
| Procedure characteristics | | | | |  |
| Concomitantly stented proximal segments | 1 | 2380 (68.9) | 2368 (67.9) | 0.73 | 0.0 |
|  | 2 | 876 (25.3) | 912 (26.1) |  |  |
|  | 3 | 185 (5.4) | 197 (5.6) |  |  |
|  | 4 | 16 (0.5) | 13 (0.4) |  |  |
| Vascular approach | A. Femoralis | 716 (20.8) | 680 (19.5) | 0.28 | 0.1 |
|  | A. Radialis | 2649 (76.8) | 2709 (77.6) |  |  |
|  | Other | 86 (2.5) | 101 (2.9) |  |  |
| Fluoroscopy time, min | | 24.6 (24.0-25.1) | 23.2 (22.6-23.9) | 0.002 | 0.0 |
| Contrast volume, mL | | 205.7 (202.7-208.7) | 187.3 (184.3-190.4) | <0.001 | 0.0 |
| Number of stents | 1 | 1310 (37.9) | 1190 (34.1) | 0.003 | 0.0 |
|  | 2 | 996 (28.8) | 1033 (29.6) |  |  |
|  | 3 or more | 1151 (33.3) | 1267 (36.3) |  |  |
| Aortic balloon pump | | 11 (0.3) | 12 (0.3) | 0.85 | 0.0 |

^a^ Myocardial infarction

^b^ Percutaneous coronary intervention

^c^ Coronary artery bypass grafting

^d^ Glomerular filtration rate

^e^ Chronic coronary syndrome

^f^ Non-ST elevated myocardial infarction

^g^ ST elevated myocardial infarction

^h^ Dual antiplatelet therapy

^i^ Low molecular weight heparin

^j^ Glycoprotein

# **Supplementary table 7.** Sensitivity analysis of all-cause mortality.

|  | **HR (95% CI)** | **P-value** |
| --- | --- | --- |
| Unadjusted | 0.70 (0.61–0.81) | <0.001 |
| Adjusted for previous CABG | 0.70 (0.61–0.81) | <0.001 |
| Adjusted for inclusion time | 0.70 (0.61–0.81) | <0.001 |
| Adjusted for previous CABG and inclusion time | 0.70 (0.61–0.81) | <0.001 |

Due to slight imbalance between groups in the patient level analysis, sensitivity analyses were made adjusting for variables that differed showing identical risk-estimates and p-values. HR, indicate hazard ratio; CI, indicates confidence interval; CABG, indicates Coronary artery bypass grafting.


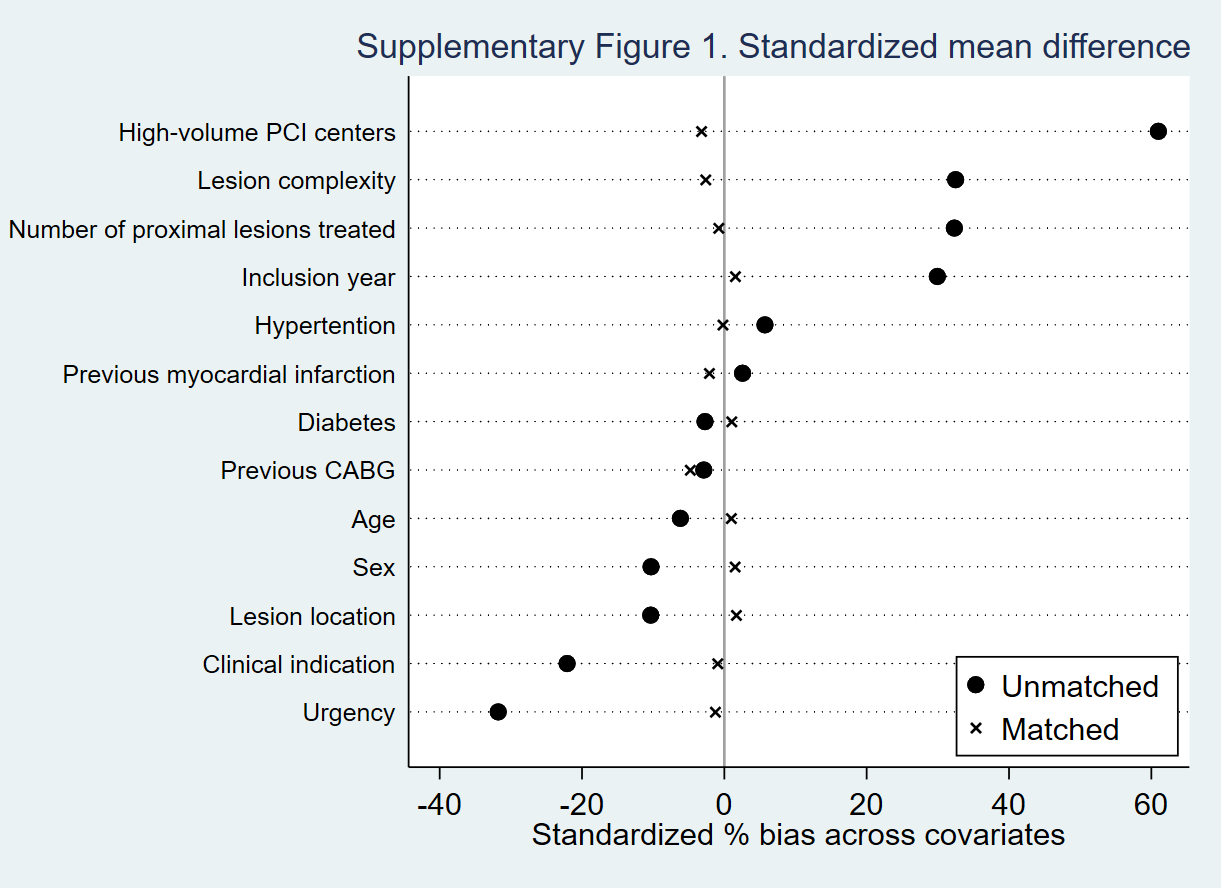


# **Supplementary figure 1.** Standardized mean differences from propensity score matching.

# **References**

1. Jakabčin J, Špaček R, Bystroň M, et al. Long-term health outcome and mortality evaluation after invasive coronary treatment using drug eluting stents with or without the IVUS guidance. Randomized control trial. HOME DES IVUS. *Catheterization and Cardiovascular Interventions* 2010; **75**(4): 578-83.

2. Kim SH, Kim YH, Kang SJ, et al. Long-term outcomes of intravascular ultrasound-guided stenting in coronary bifurcation lesions. *Am J Cardiol* 2010; **106**(5): 612-8.

3. Kim JS, Hong MK, Ko YG, et al. Impact of intravascular ultrasound guidance on long-term clinical outcomes in patients treated with drug-eluting stent for bifurcation lesions: data from a Korean multicenter bifurcation registry. *Am Heart J* 2011; **161**(1): 180-7.

4. Claessen BE, Mehran R, Mintz GS, et al. Impact of intravascular ultrasound imaging on early and late clinical outcomes following percutaneous coronary intervention with drug-eluting stents. *JACC Cardiovasc Interv* 2011; **4**(9): 974-81.

5. Zhang Y, Farooq V, Garcia-Garcia HM, et al. Comparison of intravascular ultrasound versus angiography-guided drug-eluting stent implantation: a meta-analysis of one randomised trial and ten observational studies involving 19,619 patients. *EuroIntervention* 2012; **8**(7): 855-65.

6. Wakabayashi K, Lindsay J, Laynez-Carnicero A, et al. Utility of Intravascular Ultrasound Guidance in Patients Undergoing Percutaneous Coronary Intervention for Type C Lesions. *Journal of Interventional Cardiology* 2012; **25**(5): 452-9.

7. Prati F, Di Vito L, Biondi-Zoccai G, et al. Angiography alone versus angiography plus optical coherence tomography to guide decision-making during percutaneous coronary intervention: the Centro per la Lotta contro l'Infarto-Optimisation of Percutaneous Coronary Intervention (CLI-OPCI) study. *EuroIntervention* 2012; **8**(7): 823-9.

8. Kim J-S, Kang T-S, Mintz GS, et al. Randomized Comparison of Clinical Outcomes Between Intravascular Ultrasound and Angiography-Guided Drug-Eluting Stent Implantation for Long Coronary Artery Stenoses. *JACC: Cardiovascular Interventions* 2013; **6**(4): 369-76.

9. Yoon YW, Shin S, Kim BK, et al. Usefulness of intravascular ultrasound to predict outcomes in short-length lesions treated with drug-eluting stents. *Am J Cardiol* 2013; **112**(5): 642-6.

10. Hur SH, Kang SJ, Kim YH, et al. Impact of intravascular ultrasound-guided percutaneous coronary intervention on long-term clinical outcomes in a real world population. *Catheter Cardiovasc Interv* 2013; **81**(3): 407-16.

11. Chieffo A, Latib A, Caussin C, et al. A prospective, randomized trial of intravascular-ultrasound guided compared to angiography guided stent implantation in complex coronary lesions: the AVIO trial. *Am Heart J* 2013; **165**(1): 65-72.

12. Klersy C, Ferlini M, Raisaro A, et al. Use of IVUS guided coronary stenting with drug eluting stent. *International Journal of Cardiology* 2013; **170**(1): 54-63.

13. Chen S-L, Ye F, Zhang J-J, et al. Intravascular ultrasound-guided systematic two-stent techniques for coronary bifurcation lesions and reduced late stent thrombosis. *Catheterization and Cardiovascular Interventions* 2013; **81**(3): 456-63.

14. Park KW, Kang SH, Yang HM, et al. Impact of intravascular ultrasound guidance in routine percutaneous coronary intervention for conventional lesions: data from the EXCELLENT trial. *Int J Cardiol* 2013; **167**(3): 721-6.

15. Witzenbichler B, Maehara A, Weisz G, et al. Relationship Between Intravascular Ultrasound Guidance and Clinical Outcomes After Drug-Eluting Stents. *Circulation* 2014; **129**(4): 463-70.

16. Ahn JM, Kang SJ, Yoon SH, et al. Meta-analysis of outcomes after intravascular ultrasound-guided versus angiography-guided drug-eluting stent implantation in 26,503 patients enrolled in three randomized trials and 14 observational studies. *Am J Cardiol* 2014; **113**(8): 1338-47.

17. Hong SJ, Kim BK, Shin DH, et al. Usefulness of intravascular ultrasound guidance in percutaneous coronary intervention with second-generation drug-eluting stents for chronic total occlusions (from the Multicenter Korean-Chronic Total Occlusion Registry). *Am J Cardiol* 2014; **114**(4): 534-40.

18. Jang JS, Song YJ, Kang W, et al. Intravascular ultrasound-guided implantation of drug-eluting stents to improve outcome: a meta-analysis. *JACC Cardiovasc Interv* 2014; **7**(3): 233-43.

19. Hernandez JM, Alonso JAB, Hospital JAG, et al. Clinical Impact of Intravascular Ultrasound Guidance in Drug-Eluting Stent Implantation for Unprotected Left Main Coronary Disease Pooled Analysis at the Patient-Level of 4 Registries. *Jacc-Cardiovasc Inte* 2014; **7**(3): 244-54.

20. Gao X-F, Kan J, Zhang Y-J, et al. Comparison of one-year clinical outcomes between intravascular ultrasound-guided versus angiography-guided implantation of drug-eluting stents for left main lesions: a single-center analysis of a 1,016-patient cohort. *Patient Preference and Adherence* 2014: 1299.

21. Hong S-J, Kim B-K, Shin D-H, et al. Effect of Intravascular Ultrasound–Guided vs Angiography-Guided Everolimus-Eluting Stent Implantation. *JAMA* 2015; **314**(20): 2155.

22. Tian NL, Gami SK, Ye F, et al. Angiographic and clinical comparisons of intravascular ultrasound- versus angiography-guided drug-eluting stent implantation for patients with chronic total occlusion lesions: two-year results from a randomised AIR-CTO study. *EuroIntervention* 2015; **10**(12): 1409-17.

23. Kim B-K, Shin D-H, Hong M-K, et al. Clinical Impact of Intravascular Ultrasound–Guided Chronic Total Occlusion Intervention With Zotarolimus-Eluting Versus Biolimus-Eluting Stent Implantation. *Circulation: Cardiovascular Interventions* 2015; **8**(7): e002592.

24. Zhang Y-J, Pang S, Chen X-Y, et al. Comparison of intravascular ultrasound guided versus angiography guided drug eluting stent implantation: a systematic review and meta-analysis. *BMC Cardiovascular Disorders* 2015; **15**(1).

25. Wijns W, Shite J, Jones MR, et al. Optical coherence tomography imaging during percutaneous coronary intervention impacts physician decision-making: ILUMIEN I study. *European Heart Journal* 2015; **36**(47): 3346-55.

26. Tan Q, Wang Q, Liu D, Zhang S, Zhang Y, Li Y. Intravascular ultrasound-guided unprotected left main coronary artery stenting in the elderly. *Saudi Medical Journal* 2015; **36**(5): 549-53.

27. Elgendy IY, Mahmoud AN, Elgendy AY, Bavry AA. Outcomes With Intravascular Ultrasound-Guided Stent Implantation. *Circulation: Cardiovascular Interventions* 2016; **9**(4): e003700.

28. Jian-Qi Zhang RS, W. Pang, Qiong Guo, Yingding Xu, Juan Zhang, Q. Yang, Y. Li, Jinping Mei, T. Jiang, Yu-Ming Li. Application of intravascular ultrasound in stent implantation for small coronary arteries. *J Clin Invasive Cardiol* 2016; **3**: 2-8.

29. Patel Y, Depta JP, Patel JS, et al. Impact of intravascular ultrasound on the long-term clinical outcomes in the treatment of coronary ostial lesions. *Catheterization and Cardiovascular Interventions* 2016; **87**(2): 232-40.

30. Nakatsuma K, Shiomi H, Morimoto T, et al. Intravascular Ultrasound Guidance vs. Angiographic Guidance in Primary Percutaneous Coronary Intervention for ST-Segment Elevation Myocardial Infarction - Long-Term Clinical Outcomes From the CREDO-Kyoto AMI Registry. *Circ J* 2016; **80**(2): 477-84.

31. Shin DH, Hong SJ, Mintz GS, et al. Effects of Intravascular Ultrasound-Guided Versus Angiography-Guided New-Generation Drug-Eluting Stent Implantation: Meta-Analysis With Individual Patient-Level Data From 2,345 Randomized Patients. *JACC Cardiovasc Interv* 2016; **9**(21): 2232-9.

32. Steinvil A, Zhang YJ, Lee SY, et al. Intravascular ultrasound-guided drug-eluting stent implantation: An updated meta-analysis of randomized control trials and observational studies. *Int J Cardiol* 2016; **216**: 133-9.

33. Ali ZA, Maehara A, Généreux P, et al. Optical coherence tomography compared with intravascular ultrasound and with angiography to guide coronary stent implantation (ILUMIEN III: OPTIMIZE PCI): a randomised controlled trial. *The Lancet* 2016; **388**(10060): 2618-28.

34. Meneveau N, Souteyrand G, Motreff P, et al. Optical Coherence Tomography to Optimize Results of Percutaneous Coronary Intervention in Patients with Non–ST-Elevation Acute Coronary Syndrome. *Circulation* 2016; **134**(13): 906-17.

35. Sheth TN, Kajander OA, Lavi S, et al. Optical Coherence Tomography–Guided Percutaneous Coronary Intervention in ST-Segment–Elevation Myocardial Infarction. *Circulation: Cardiovascular Interventions* 2016; **9**(4).

36. Bavishi C, Sardar P, Chatterjee S, et al. Intravascular ultrasound-guided vs angiography-guided drug-eluting stent implantation in complex coronary lesions: Meta-analysis of randomized trials. *Am Heart J* 2017; **185**: 26-34.

37. Qian C, Feng H, Cao J, Zhang G, Wang Y. Intravascular ultrasound guidance in drug-eluting stents implantation: a meta-analysis and trial sequential analysis of randomized controlled trials. *Oncotarget* 2017; **8**(35): 59387-96.

38. Buccheri S, Franchina G, Romano S, et al. Clinical Outcomes Following Intravascular Imaging-Guided Versus Coronary Angiography–Guided Percutaneous Coronary Intervention With Stent Implantation. *JACC: Cardiovascular Interventions* 2017; **10**(24): 2488-98.

39. Nerlekar N, Cheshire CJ, Verma KP, et al. Intravascular ultrasound guidance improves clinical outcomes during implantation of both first- and second generation drug-eluting stents: a meta-analysis. *Eurointervention* 2017; **12**(13): 1632-42.

40. Iannaccone M, D'Ascenzo F, Frangieh AH, et al. Impact of an optical coherence tomography guided approach in acute coronary syndromes: A propensity matched analysis from the international FORMIDABLE-CARDIOGROUP IV and USZ registry. *Catheterization and Cardiovascular Interventions* 2017; **90**(2): E46-E52.

41. Andell P, Karlsson S, Mohammad MA, et al. Intravascular Ultrasound Guidance Is Associated With Better Outcome in Patients Undergoing Unprotected Left Main Coronary Artery Stenting Compared With Angiography Guidance Alone. *Circulation: Cardiovascular Interventions* 2017; **10**(5): e004813.

42. Kim YH, Her A-Y, Rha S-W, et al. Three-Year Major Clinical Outcomes of Angiography-Guided Single Stenting Technique in Non-Complex Left Main Coronary Artery Diseases. *International Heart Journal* 2017; **58**(5): 704-13.

43. Tian J, Guan C, Wang W, et al. Intravascular Ultrasound Guidance Improves the Long-term Prognosis in Patients with Unprotected Left Main Coronary Artery Disease Undergoing Percutaneous Coronary Intervention. *Sci Rep* 2017; **7**(1): 2377.

44. Ye Y, Yang M, Zhang S, Zeng Y. Percutaneous coronary intervention in left main coronary artery disease with or without intravascular ultrasound: A meta-analysis. *PLOS ONE* 2017; **12**(6): e0179756.

45. Zhang J, Gao X, Kan J, et al. Intravascular Ultrasound Versus Angiography-Guided Drug-Eluting Stent Implantation: The ULTIMATE Trial. *J Am Coll Cardiol* 2018; **72**(24): 3126-37.

46. Maehara A, Mintz GS, Witzenbichler B, et al. Relationship Between Intravascular Ultrasound Guidance and Clinical Outcomes After Drug-Eluting Stents. *Circulation: Cardiovascular Interventions* 2018; **11**(11).

47. Smilowitz NR, Mohananey D, Razzouk L, Weisz G, Slater JN. Impact and trends of intravascular imaging in diagnostic coronary angiography and percutaneous coronary intervention in inpatients in the United States. *Catheterization and Cardiovascular Interventions* 2018; **92**(6): E410-E5.

48. Wang Y, Mintz GS, Gu Z, et al. Meta-analysis and systematic review of intravascular ultrasound versus angiography-guided drug eluting stent implantation in left main coronary disease in 4592 patients. *BMC Cardiovascular Disorders* 2018; **18**(1).

49. Choi KH, Song YB, Lee JM, et al. Impact of Intravascular Ultrasound-Guided Percutaneous Coronary Intervention on Long-Term Clinical Outcomes in Patients Undergoing Complex Procedures. *JACC Cardiovasc Interv* 2019; **12**(7): 607-20.

50. Kim N, Lee JH, Jang SY, et al. Intravascular modality‐guided versus angiography‐guided percutaneous coronary intervention in acute myocardial infarction. *Catheterization and Cardiovascular Interventions* 2020; **95**(4): 696-703.

51. Darmoch F, Alraies MC, Al‐Khadra Y, Moussa Pacha H, Pinto DS, Osborn EA. Intravascular Ultrasound Imaging–Guided Versus Coronary Angiography–Guided Percutaneous Coronary Intervention: A Systematic Review and Meta‐Analysis. *Journal of the American Heart Association* 2020; **9**(5).

52. Hong SJ, Mintz GS, Ahn CM, et al. Effect of Intravascular Ultrasound-Guided Drug-Eluting Stent Implantation: 5-Year Follow-Up of the IVUS-XPL Randomized Trial. *JACC Cardiovasc Interv* 2020; **13**(1): 62-71.

53. Iannaccone M, Abdirashid M, Annone U, et al. Comparison between functional and intravascular imaging approaches guiding percutaneous coronary intervention: A network meta‐analysis of randomized and propensity matching studies. *Catheterization and Cardiovascular Interventions* 2020; **95**(7): 1259-66.

54. Park H, Ahn J-M, Kang D-Y, et al. Optimal Stenting Technique for Complex Coronary Lesions. *JACC: Cardiovascular Interventions* 2020; **13**(12): 1403-13.

55. Ladwiniec A, Walsh SJ, Holm NR, et al. Intravascular ultrasound to guide left main stem intervention: a NOBLE trial substudy. *EuroIntervention* 2020; **16**(3): 201-9.

56. Kinnaird T, Johnson T, Anderson R, et al. Intravascular Imaging and 12- Month Mortality After Unprotected Left Main Stem PCI: An Analysis From the British Cardiovascular Intervention Society Database. *JACC: Cardiovascular Interventions* 2020; **13**: 346-57.

57. Mentias et al. Long-Term Outcomes of Coronary Stenting With and Without Use of Intravascular Ultrasound. *J Am Coll Cardiol Intv* 2020; **13**: 1880–90

58. Gao XF, Ge Z, Kong XQ, et al. 3-Year Outcomes of the ULTIMATE Trial Comparing Intravascular Ultrasound Versus Angiography-Guided Drug-Eluting Stent Implantation. *JACC Cardiovasc Interv* 2021; **14**(3): 247-57.

59. Hong SJ, Kim D, Kim BK, et al. Acute and one-year clinical outcomes of pre-stenting intravascular ultrasound: a patient-level meta-analysis of randomised clinical trials. *EuroIntervention* 2021; **17**(3): 202-11.

60. Ali ZA, Karimi Galougahi K, Maehara A, et al. Outcomes of optical coherence tomography compared with intravascular ultrasound and with angiography to guide coronary stent implantation: one-year results from the ILUMIEN III: OPTIMIZE PCI trial. *EuroIntervention* 2021; **16**(13): 1085-91.

61. Kang DY, Ahn JM, Yun SC, et al. Long-Term Clinical Impact of Intravascular Ultrasound Guidance in Stenting for Left Main Coronary Artery Disease. *Circ Cardiovasc Interv* 2021; **14**: e011011.
